# Protecting honey bees (*Apis mellifera*) from thermal stress: Probiotics and prebiotics buffer the survival and antioxidant enzyme activity

**DOI:** 10.1371/journal.pone.0352149

**Published:** 2026-07-15

**Authors:** Rahil Mirabi Moghaddam, Najmeh Sahebzadeh, Ehsan Rakhshani, Naser Tajabadi, Rassol Bahreini

**Affiliations:** 1 Department of Plant Protection, Faculty of Agriculture, University of Zabol, Zabol, Iran; 2 Department of Honey Bee, Animal Sciences Research Institute of Iran, Karaj, Iran; 3 Department of Biological Sciences, University of Alberta, Edmonton, Alberta, Canada; University of Murcia: Universidad de Murcia, SPAIN

## Abstract

Environmental temperature profoundly influences honey bee (*Apis mellifera*) physiology, behavior, and survival. In recent years, nutritional interventions have gained attention as potential strategies to mitigate stress-induced physiological disruptions in honey bees. This study evaluated the efficacy of dietary supplementations enriched with combined prebiotics and probiotics in alleviating temperature-related stress. Newly-emerged worker bees were first maintained under optimal conditions (35 °C) and received dietary supplements for 21 days, after which they were exposed to four temperature regimes (4, 15, 35, and 40 °C) to evaluate their stress resilience. Bee survival and the activity of key antioxidant enzymes (i.e., superoxide dismutase, ascorbate peroxidase, catalase, and peroxidase) were assessed. Bees received a commercial probiotic (Progen®) and the prebiotic Inulin in equal proportions at concentrations of 2.5, 5, 7.5, and 10 g L^-1^. The results demonstrated that bees exposed to thermal stress and supplemented with the highest combined dose of probiotic and prebiotic (10 g L^-1^ of each supplement) exhibited significantly higher survival compared with bees receiving lower doses (2.5 g L^-1^), or an unsupplemented diet. In unsupplemented control groups, temperature stress induced marked increases in antioxidant enzyme activities, indicating significant oxidative stress. In contrast, supplementation resulted in a dose-dependent reduction in enzyme activities across all temperatures, strongly suggesting a protective, and stress-buffering effect. These physiological benefits were accompanied by an increase in gut microbial abundance and enhanced indicators of energy metabolism in the supplemented bees. Overall, our findings demonstrated that combined probiotic and prebiotic supplementation can effectively buffer honey bees against temperature-induced physiological stress, highlighting a practical nutritional strategy to enhance colony resilience under the fluctuating environmental conditions associated with seasonal variation and ongoing global climate change.

## Introduction

The Apidae family includes a diverse array of solitary, social, domesticated, and managed bee species that engage in pollination nearly three-quarters of the world’s flowering plant species, critical to food production [1]. Among these species, the European honey bee, *Apis mellifera* L. (Hymenoptera: Apidae), has an evolutionary history spanning at least 30 million years and, due to human-mediated dispersal, is now found in nearly every country worldwide [2]. However, certain honey bee populations have experienced fluctuations and declines in recent decades [3,4]. Several factors can challenge colony resilience, including parasites, pathogens, agrochemicals, and environmental thermal stresses [5–7]. Honey bees (*A. mellifera*) rely on meticulous thermoregulation, through fanning, evaporation, and metabolic heat, to maintain a stable in-hive environment—typically around 34°C to 36°C—which is indispensable for optimal brood development, immune function, and overall colony fitness [8]. However, when environmental temperature fluctuations exceed the colony’s behavioral and physiological capacity to regulate heat, individual bees are subjected to severe thermal stress [9]. This loss of thermal homeostasis triggers a cascade of detrimental cellular events, most notably an imbalance between the generation of reactive oxygen species (ROS) and the insect’s endogenous antioxidant defense mechanisms, culminating in severe cellular oxidative stress [10,11]. Disrupting this homeostasis leads to severe consequences, including increased sensitivity to pesticides, impaired behavioral and cognitive functions, and disorganized brain synapses [12–15]. Ultimately, thermal stress undermines vital biological processes, from immune function and foraging to reproductive success [16]. Such disruptions in colony homeostasis may threaten colony health, potentially leading to increased mortality under severe conditions [17–19]. Under these challenging conditions, the bee immune systems are highly vulnerable, underscoring the critical need to investigate mechanisms that activate honey bee defense systems. The defensive responses of honey bees against foreign and pathogenic factors encompass, integrating a complex network of interrelated systems, including antimicrobial peptides, hemagglutinin, phenoloxidase activity, and the antioxidant system (AOS) [20]. Consequently, stable temperature maintenance is critical to bee survival. As global climates become increasingly erratic, exploring dietary interventions—such as probiotics and prebiotics—that can buffer these physiological stressors and preserve vital antioxidant enzyme activity under thermal extremes has become a pressing priority for sustaining apicultural health. While beekeeping practices and nutritional support often mitigate these impacts in managed bee colonies, extreme environmental conditions remain a significant stressor [21].

Oxidative stress is a pivotal process with potentially severe adverse effects in eukaryotic organisms. Reactive oxygen species (ROS), generated during normal metabolic activity, play a key role in oxidative stress. To mitigate or counteract ROS-mediated oxidative stress, insects employ enzymatic mechanisms that facilitate oxidative inactivation such as superoxide dismutase, catalase, and peroxidase, or enable intracellular ROS scavenging through glutathione peroxidase and glutathione reductase [22,23]. A primary enzyme responsible for hydrogen peroxide neutralization, catalase, is particularly vulnerable to external stimuli, including elevated temperatures, chemical exposure, insecticides, and interactions with certain plant compounds [24], making it a valuable indicator of the overall status of antioxidant systems [23–25]. To protect bees against environmental threats such as thermal stress, environmental pollution, and agrochemicals, it is essential to understand how their antioxidant system is activated and which factors enhance or impair its function. Among the various factors that strengthen the antioxidant system, moderate environmental conditions, antioxidant-rich nutrition, natural physiological activities, and the use of nutritional supplements can be considered. Among these approaches, feeding bees with probiotics and prebiotics strengthens the gut microbiome and improves the organism’s ability to withstand oxidative stress [26].

A diverse group of insects, including honey bees, harbor beneficial symbiotic microorganisms that are crucial for maintaining proper gastrointestinal function [27]. These microorganisms (i.e., probiotics) contribute to carbohydrate fermentation and produce essential vitamins and amino acids for the host [27]. Probiotics are live microorganisms that naturally occur in small quantities within the honey bee’s gut. They enhance the proliferation of beneficial microbes while suppressing pathogenic species, thereby preventing or treating gut microbial dysbiosis resulting from diseases or indiscriminate antibiotic use [28]. Alternatively, prebiotics are non-digestible food components that induce specific alterations in the composition or activity of the gastrointestinal microbiota, promoting their population growth [29]. Beyond stimulating beneficial gut bacteria, prebiotics can also inhibit the growth of harmful and potentially pathogenic microorganisms in the intestine of honey bees [30]. Recently, prebiotics have been utilized to enhance honey bee survival against pathogens such as *Nosema* spp. [28]. Key prebiotics include inulin, lactulose, xylan, fiber gums, xylo-oligosaccharides, fructo-oligosaccharides, and lactitol [31]. Empirical investigations into the effects of commercial probiotics and prebiotics on honey bee health have yielded variable outcomes. For instance, probiotic supplementation has been shown to significantly affect honey bee colony strength, wax gland development, honey production, and disease resistance [32]. The nutritional supplementation of queen larvae and young queens with specific probiotic compounds, can exert an influence on the development of the hypopharyngeal and mandibular glands. This enhanced glandular development, in turn, culminates in the superior production of royal jelly and a more robust pheromonal profile, both of which are critical to the maintenance of colony integrity and coherence [33]. Certain probiotics also exhibit antibacterial and anti-inflammatory properties, and can positively modulate innate immunity, cellular responses, and cytokine profiles [34,35]. Despite these findings, uncertainty remains as to whether probiotics and their synergistic compounds, such as prebiotics, can mitigate the toxic effects of pesticides and other environmental stressors on honey bees.

Thermal fluctuations, ranging from extreme heat waves to prolonged cold spells, act as significant stressors that restructure the honeybee gut microbiome, ultimately dictating the colony’s physiological resilience. Under heat stress, the delicate balance of the gut community is disrupted; research by Van Wyk et al. [34] demonstrates that high temperatures cause distinct ecological shifts that can exacerbate the impact of co-occurring pathogen infections. These shifts are often driven by the narrow thermal niches of specialized gut symbionts, which vary in their sensitivity to heat [36]. Conversely, cold stress during wintering induces a different microbial signature characterized by a high abundance of *Lactobacilli* spp. [35]. This seasonal transition in the hindgut microbiota is influenced by both worker age and the ambient climate [37], resulting in a community structure that differs fundamentally from that of summer bees [38]. The stability of these microbial communities is vital for colony health; a resilient microbiome functions as a metabolic engine and a protective barrier, bolstering the bees’ ability to withstand thermal extremes and reducing pathogen susceptibility. To maintain this internal equilibrium, bees employ behavioral and physiological mechanisms, while the microbiome, in turn, produces metabolites that support the host’s immune system. When these natural defenses are strained by climate instability, targeted interventions such as probiotics and prebiotics offer a promising solution. By directly seeding beneficial bacteria or indirectly providing the substrates necessary to fuel their growth, these supplements can stabilize the gut environment, reinforcing the bee’s natural resilience against the metabolic and immunological tolls of thermal stress.

Environmental temperature fluctuations, including increasingly frequent heat waves and cold spells, are known to profoundly reshape the honey bee gut microbiome. Such thermal stress can lead to dysbiosis, favoring thermotolerant taxa while reducing microbial diversity, which in turn increases pathogen susceptibility [34,36]. While core symbionts like *Lactobacillus*spp. play a key role in maintaining host health during winter and seasonal transitions [35,37,38], the mechanisms by which these microbial shifts influence physiological resilience remain a critical area of study. Although the present research focuses on physiological and antioxidant parameters rather than direct microbial profiling, understanding these interactions is essential for developing effective management strategies. Therefore, this study aims to evaluate how synbiotic (probiotic + prebiotic) supplementation might mitigate the negative impacts of thermal stress, potentially by supporting the host’s internal homeostasis and microbial balance.

## Materials and methods

### Insects and exposure protocols

This research was conducted from March 2024 to September 2024 (encompassing the spring and summer seasons) at the Honey bee laboratory of the Department of Plant Protection, University of Zabol (31.0363° N, 61.4892° E), Sistan and Baluchestan province, Iran. To obtain newly-emerged bees, sealed brood frames were selected from healthy *Apis mellifera meda* Skorikov colonies. These colonies showed no visible signs of *Varroa destructor* Anderson & Trueman, and *Nosema ceranae* Fries, which were assessed using established protocols [39].

To collect newly-emerged worker bees, the sealed brood frames were maintained in a germinator (600ax, Noor Sanat Azma, Iran) at 35 ± 1 °C and 60% RH, in the dark [40]. To standardize the age of the cohort of honey bees, only those that emerged from the sealed brood frames within the first 24 hours were collected. The newly-emerged worker bees were maintained in the germinator under the aforementioned conditions and provided *ad libitum* access to 50% sucrose syrup and water [40]. Groups of 100 newly emerged worker bees were placed in plastic cages (35 × 15 × 15 cm) (S1 Fig). The cages were made of plastic netting to allow visibility and airflow, and they had secure lids with two 50 mL gravity feeder containers for easy access. The cages were labeled and randomly placed in the germinator. Two 50-mL plastic bottles, one for water and another for sucrose syrup 50% with each dietary supplement, were inverted on the cage lids. The openings of the filled bottles were sealed with two layers of Parafilm™, and small holes were made using an entomology pin to allow honey bees to feed. In addition to syrup and water, a pollen diet was provided *ad libitum* using a custom-designed delivery system. Medical syringes were modified by removing their front tips to create an open cylindrical feeder. These syringes were filled with pollen paste and inserted horizontally into the side walls of each cage, allowing the bees free access to the diet while ensuring no leakage or escape (S1 Fig). Food and water were replenished daily, and dead bees were recorded and removed daily.

### Preparation of dietary supplements

A dietary supplement was prepared using a commercial probiotic, Progen (Tech-Gene Company, Tehran, Iran). According to the manufacturer, Progen® is a multi-strain probiotic containing *Lactobacillus acidophilus*, *Lactobacillus casei*, *Lactobacillus plantarum*, *Bifidobacterium bifidum*, *Enterococcus faecium*, and *Bacillus subtilis* (at a minimum concentration of 2 × 10⁹ CFU g ⁻ ¹). This was used in combination with the prebiotic Inulin (Sensus Company, New Zealand), which is a long-chain fructan extracted from chicory root. Treatments included *2.5p + p* (2.5 g L^-1^ probiotic+2.5 g L^-1^ prebiotic), *5p + p* (5 g L^-1^ probiotic +5 g L^-1^ prebiotic), *7.5p + p* (7.5 g L^-1^ probiotic+7.5 g L^-1^ prebiotic), and *10p + p* (10 g L^-1^ probiotic +10 g L^-1^ prebiotic). A negative control group was maintained on 50% sucrose syrup without probiotic or prebiotic treatments.

### Effects of probiotics and prebiotics under temperature stress

Newly emerged honey bees (1-day-old) were fed a combination of various concentrations of Progen probiotic and Inulin prebiotic over a 21-day period [41]. Following this period, bees were subjected to distinct thermal stress conditions (4, 15, and 40 °C) for up to 10 days, or until total mortality occurred, while 35 °C served as the reference control. Temperature stress treatments were carried out in a germinator with 60% R.H. Each treatment was repeated five times with 100 honey bee individuals (500 honey bees per treatment in total).

### Survival monitoring

At the end of the 21-day supplementation period, thermal stress treatments were initiated. From each bioassay cage, 50 live honey bees were randomly selected and transferred into individually labeled 1 L plastic chambers for survival monitoring under the assigned temperature regimes. The remaining live bees were immediately utilized for biochemical assessments. To ensure continuous nourishment during the stress challenge, each survival chamber was fitted with two 50 mL plastic bottle feeders, providing *ad libitum* access to 50% sucrose solution and water. The chambers were maintained in a germinator at 35 °C and 60% RH, in darkness, and were examined daily for bee mortality over a 10-day period [40]. Bees were classified as deceased if they were observed lying on their sides and exhibited no antennal or abdominal movement upon stimulation [42].

### Biochemical assays

#### Tissue homogenization.

Honey bees from both treatment and control groups were anesthetized using the chilling method. From each replicate (i.e., cage), 50 live honey bees were randomly selected, and their heads and thoraxes were separated using a sharp sterile blade. For each sample, the tissues were homogenized with a micro pestle (Microteknik, India) in an ice-cold isolation buffer (0.01 M Tris-HCl buffer). Samples were centrifuged at 13,000 g using an Eppendorf 5810R centrifuge (Eppendorf AG, Hamburg, Germany). The supernatants were collected and stored at −80 °C for subsequent experiments [43].

#### Catalase assay.

Catalase activity (CAT) was determined according to Aebi [44]. A reaction mixture containing 50 μL of honey bee tissue homogenate and 500 μL of 1% hydrogen peroxide was incubated at 28 °C for 10 minutes. The absorbance at 240 nm was subsequently measured and reported as ΔA240 nm/min/mg protein using a spectrophotometer (S-2150, Unico, Shanghai).

#### Superoxide dismutase assay.

To measure the superoxide dismutase activity (SOD), a xanthine oxidase solution was prepared and added to 2 mL of 0.1 M phosphate buffer (pH 7). Then, 60 μL of this mixture was added to a solution containing nitro blue tetrazolium and xanthine, both dissolved in 0.1 M phosphate-buffered saline (pH 7). Finally, 20 μL of the honey bee tissue homogenate was added to the entire mixture, and incubation was initiated in the dark for 20 minutes. The absorbance was read at 560 nm and reported as ΔA560 nm/min/mg protein using a spectrophotometer (S-2150, Unico, Shanghai) [45].

#### Peroxidase assay.

Peroxidase (POX) activity was quantified following the methodology described by Chaoui et al. [46]. The reaction mixture, with a total volume of 3 mL, consisted of 50 mM phosphate buffer, 0.2 mM guaiacol, 10 mM hydrogen peroxide (H_2_O_2_), and the balance made up of distilled water. The enzymatic reaction was initiated by the addition of 50 µg of protein, which was extracted from 10 µl hemolymph. The increase in absorbance at 470 nm was monitored to quantify peroxidase activity using a spectrophotometer (S-2150, Unico, Shanghai). Enzyme activity was expressed as the change in absorbance per minute per milligram of protein.

#### Ascorbate peroxidase assay.

Ascorbate peroxidase (APX) activity was measured following the method of Asada [47]. Fifty microliters of honey bee tissue homogenate were added to a mixture containing 150 μL of 100 mM phosphate buffer and 2.5 mM ascorbic acid. Two hundred microliters of hydrogen peroxide were then introduced, and the absorbance was monitored at 290 nm for 5 minutes. The results were expressed as ΔA290 nm/min/mg protein using a spectrophotometer (S-2150, Unico, Shanghai).

### Statistical analysis

All statistical analyses were conducted using SPSS software (version 27.0) and GraphPad Prism (version 10.6.1). For the survival analysis, the Kaplan–Meier estimator was applied to calculate survival probabilities, and the Log-rank test was used to compare survival curves between treatments. Biochemical parameters, including the activities of catalase (CAT), superoxide dismutase (SOD), ascorbate peroxidase (APX), and peroxidase (POX), as well as total protein content, were analyzed using a 4 × 5 factorial experiment based on a completely randomized design (CRD) with five replicates per treatment. The experimental factors included dietary supplements (five levels) and exposure temperatures (four levels). Biochemical data were subjected to two-way analysis of variance (ANOVA), followed by Tukey’s HSD post-hoc test. To satisfy the assumptions of normality, mortality proportions were arcsine-transformed before analysis; however, all data are presented as untransformed means for clarity. Data visualization was performed using GraphPad Prism.

## Results

### Dietary supplements and survival under thermal stress

**Fig 1:**
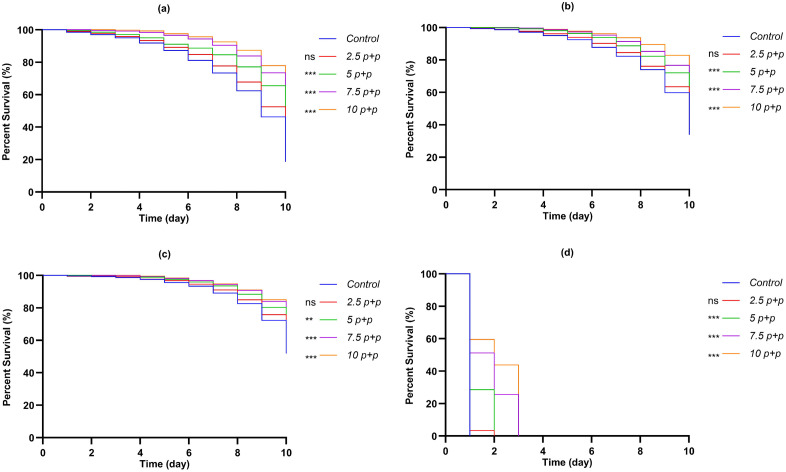
Kaplan-Meier survival curves of honey bees over 10 days. Bees were exposed to different combinations of dietary supplements (probiotics and prebiotics) and temperatures (a: 4 °C, b: 15 °C, c: 35 °C, and d: 40 °C). Statistical differences were determined by comparing each treatment group with the control group. Significant levels are indicated as follows: ns (not significant, *p* > 0.05), ** (*p* < 0.01), and *** (*p* < 0.0001).

The Kaplan-Meier survival curves indicated an overall significant survival distribution of honey bees over a 10-day period, where exposed to different concentrations of probiotic and prebiotic (i.e., 0, 2.5, 5, 7.5, and 10 g L^-1^), and to various thermal stress conditions (i.e., 4, 15, 35, and 40 °C) (Fig 1).

### Thermal stress

The survival probability of honey bees decreased across all groups, indicating the negative impact of cold stress at 4 °C on bee survival (Fig 1a). The survival probability of the control group experienced a more rapid decline in survival compared to the other treatments, especially later in the experimental period. This indicates that the combination of probiotic and prebiotic supplements ≥ *5p + p* had a positive effect on the survival of honey bees under cold stress at 4 °C (χ^2^ = 129.9, df = 4, *p* < 0.0001). The results showed that groups with higher doses of probiotics and prebiotics (i.e., *7.5p + p-4°C*, and *10p + p-4°C*) exhibited a significantly greater survival (>70%) compared to lower doses (i.e., *2.5p + p*,4 °C and *5p + p*,4 °C).

As shown in Fig 1b, the survival rate was generally higher at 15 °C thermal stress than at 4 °C, but rates still decreased over time. Similar to the 4 °C results, treatment with probiotics and prebiotics significantly improved the survival rate compared to the control group (χ^2^ = 59.24; df = 4, *p* < 0.0001). The treatment *10p + p* at 15 °C treatment maintained the highest survival level (>80%) at the end of the experimental period.

The survival rate of all groups when bees were exposed to an optimal temperature (35 °C), was significantly higher (>70%) for all dietary supplements, throughout the 10-day period compared to the other temperatures studied at 4, 15, or 40 °C (χ^2^ = 24.88, df = 4, *p* < 0.0001) (Fig 1c). Honey bee survival exhibited pronounced stability at the thermally optimal temperature of 35 °C. The Kaplan–Meier plot at 35 °C (Fig 1c) shows that during the early phase of the experiment (approximately days 0–6), survival rates for all treatments, including the control, remained exceptionally high (≈100%) with no detectable decline. Furthermore, at this optimal temperature, the difference between the survival curves for the groups that received nutritional supplements (between 2.5 and 10 g L^-1^) was negligible.

The results indicated that severe heat stress (i.e., 40 °C) rapidly reduced bee survival (Fig 1d) as a lethal environmental condition. The slope of the survival curves in all groups at 40 °C, declined sharply by the third day of the trial. The honey bees in the control group and those supplemented with the *2.5p + p* nutritional blend exhibited mortality rates of 100% and 96%, respectively, during early exposure to thermal stress at 40°C thermal stress (on day 1). Conversely, bees consuming the *5p + p* combination exhibited an increase in survival duration by one additional day. Survival was further prolonged to two additional days in the 7.*5p + p* group, with the highest concentration (*10p + p* diet) yielding the longest survival time, extended by three days. A significant difference was observed between the survival curves of the different groups (χ^2^ = 112.1, df = 4, *p* < 0.0001) (Fig 1d).

According to our results (Fig 1a, 1b, and 1c), survival in all groups remained high and close to 100% during the initial phase of the experiment (day 1–4), indicating that exposure to 4, 15, and 35 °C did not cause acute, or rapid mortality. However, survival began to decline over time (starting around day 4–5), suggesting cumulative effects of thermal stress on the bees’ physiology. By the end of the experiment (day 10), the cumulative survival for the control group had decreased by 35–40% at 15 °C, whereas it was higher for the diet treated groups. In contrast, Fig 1d shows that severe heat stress (40 °C) caused rapid mortality from the beginning of the experiment.

### The antioxidant activities

**Fig 2:**
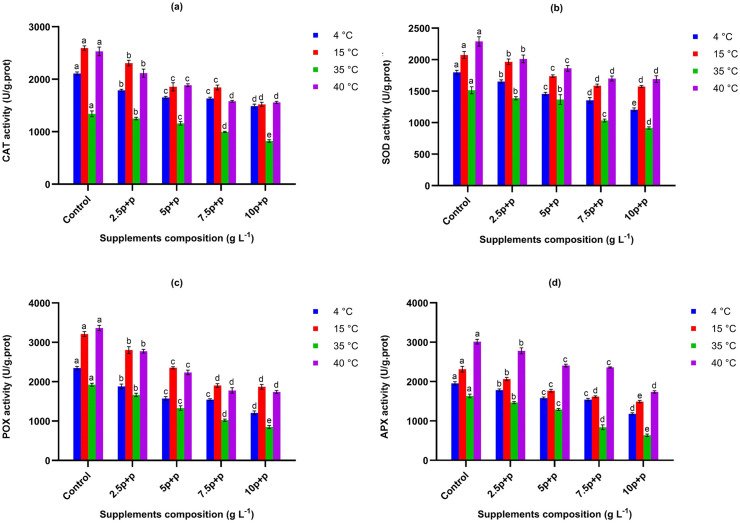
The effects of different combinations of dietary supplements (probiotics and prebiotics) and temperatures (4 °C, 15 °C, 35 °C, and 40 °C) on activity of antioxidant enzymes (a: CAT, b: SOD, c: POX, and d: APX) in honey bees. At each specified temperature, columns labeled with the same letter (a, b, c, and d) are not significantly different (*p > 0.05*).

The effects of various concentrations of probiotic and prebiotic supplements (i.e., 2.5, 5, 7.5, and 10 g L^-1^) on the activity of four antioxidant enzymes: catalase (CAT), superoxide dismutase (SOD), peroxidase (POX), and ascorbate peroxidase (APX) in honey bees exposed to different temperatures (i.e., 4, 15, 35, and 40 °C) were evaluated (Fig 2).

### Catalase activity

The impact of various combinations of probiotic and prebiotic supplements on the catalase (CAT) activity of honey bees subjected to different temperatures (Fig 2a). The highest CAT activity (2594.61 U/g protein) was recorded in the control group at temperature of 15 °C. Conversely, the lowest activity (823.11 U/g protein) was occurred at *10p + p-35°C*, which corresponds to the honey bee colony’s optimal homeostatic temperature. A clear, dose-dependent inverse relationship was observed between supplement concentrations and CAT activity. Across all tested temperatures, particularly at the stressful extremes of 15 and 40 °C, CAT activity was significantly reduced in all supplemented groups compared to the control (F = 228.497, df = 12, *p* < 0.0001) (Fig 2a). Specifically, the *10p + p* dosage demonstrated the most consistent reduction in CAT activity across most temperature regimes (particularly at 4 and 40 °C) (Fig 2a).

### Superoxide dismutase activity

For all the nutritional supplements, the lowest level of SOD activity occurred at 35 °C. This temperature represents a baseline for SOD activity, as it is optimal for the honey bees’ feeding, survival, and daily activities, causing minimal thermal stress. The results demonstrated an increase in SOD activity at 4, 15, and 40 °C compared to 35 °C. For all diets, the highest SOD activity was observed at 40 °C, followed by 15 °C, and 4 °C, respectively. However, after the application of probiotic and prebiotic supplements, this activity was diminished. This trend indicated that following increasing in the concentration of the supplements from 2.5 to 10 g L^-1^, the activity of the SOD enzyme also decreased across all tested temperatures (F = 51.418, df = 12, *p* < 0.0001) (Fig 2b).

### Peroxidase activity

As seen in Fig 2c, the highest enzyme level was observed at 15 °C, while 35 °C had the lowest POX activity at all supplement concentrations. At all temperatures, an increase in supplement concentration led to a decrease in POX activity. These results suggested that the tested supplements mitigate oxidative stress in the stressed honey bees, thereby reducing the need for increased POX enzyme production in bees. As the concentration of the supplement compounds increased (from control to 10 g L^-1^), POX activity significantly decreased at all temperatures (F = 29.598, df = 12, *p* < 0.0001).

### Ascorbate peroxidase activity

The most severe oxidative stress was unequivocally demonstrated in the control group (no supplementation) at 40 °C, where Ascorbate peroxidase activity (APX) peaked at approximately 3000 U/g protein, representing the highest value across the entire dataset (Fig 2d). This finding underscores that acute thermal stress at 40 °C acted as the most potent stimulant for activating ascorbate-dependent antioxidant defense mechanisms in the honey bee. In the supplemented groups, APX activity at the lethal 40 °C temperature decreased markedly to 1738.46 with increasing supplement dosage. Specifically, the highest dosage, *10p + p*, reduced APX activity to approximately 1750 U/g protein, a major and vital reduction compared to the 3000 U/g protein observed in the control treatment (F = 108.86, df = 12, *p* < 0.0001). Furthermore, a notable protective effect was observed at the colony’s optimal homeostatic temperature of 35 °C. Here, the higher dosages (*7.5p + p* and *10p + p*) reduced APX activity to the lowest levels overall in the experiment (ranging from approximately 650–850 U/g protein).

## Discussion

We evaluated the efficacy of probiotic (i.e., Progen) and prebiotic (i.e., Inulin) supplementation across a range of temperatures (4, 15, 35, and 40 °C), providing novel insight into the role of these dietary interventions in modulating honey bee resilience. This study systematically elucidated how the magnitude and direction of thermal stress dictate considerable benefits of gut microbiome-modulating supplements. We found that exposure to a low temperature (4 °C) resulted in a significant reduction in honey bee survival, which aligns with previous empirical findings on the severe thermal stress [16]. Honey bees, despite their remarkable ability to cluster and thermoregulation in winter, are highly susceptible to prolonged exposure to temperatures below their optimal physiological range [48]. For instance, Stabentheiner et al. [49] demonstrated that sustained low temperatures lead to an increase in energy expenditure and a reduction in individual lifespan in both isolated bees and small clusters. In general, based on the results of the Kaplan-Meier survival curves, it can be concluded that probiotic and prebiotic dietary supplements exert a significant positive effect on longevity under thermal stress. Our results show that increasing the supplement dosage improves resistance to both cold and heat stress, thereby reducing mortality. Specifically, evaluating the supplement dosage to 10 g L^-1^ led to improved health and survival of honey bee colonies under simulated adverse weather conditions.

Numerous studies have demonstrated the capacity of beneficial microorganisms to enhance honey bee resilience against various environmental and biological stressors. For instance, Liu et al. [50] showed that supplementation with specific strains of *Lactobacillus* spp. and *Bifidobacterium* spp. could improve the overwintering success of honey bee colonies, which inherently involves coping with prolonged cold exposure. Similarly, Dolezal et al. [51] found that cold acclimation and diet, which influences gut health, could impact the survival of individual bees, providing a foundational baseline for our observed effects. Raymann and Moran [52] and Zheng et al. [53] extensively reviewed the critical role of the indigenous honey bee gut microbiota in nutrient processing and immune system function, suggesting that improving these systems could greatly reduce the metabolic demands caused by cold stress.

The survival dynamics of bees observed at 15 °C in our control group reflected a moderate cold stress scenario. Survival was higher than at 4 °C but still substantially lower than at 35 °C over time. These findings are consistent with studies showing that while honey bees can survive at temperatures above their chill coma threshold (typically around 8−10 °C), prolonged exposure to moderate cold increases metabolic demands, reduces activity, and ultimately shortens lifespan compared to optimal conditions [54]. Although these temperatures are not acutely lethal to bees, they impose a chronic physiological cost that gradually impacts survival [55], as our data clearly illustrate. Our survival analysis unequivocally demonstrates a significant and dose-dependent enhancement of honey bee survival when supplemented with probiotics and prebiotics under cold stress conditions, at both 4 °C (extreme cold) and 15 °C (moderate cold). The survival curves for supplemented groups consistently outperformed the respective control groups, with higher doses (i.e., 7.5 and 10 g L^-1^) exhibiting superior protective effects. This marked improvement in cold tolerance is a pivotal finding, aligning with and extending recent evidence suggesting the critical role of a robust gut microbiome in honey bee health and stress physiology [33,56].

Probiotic supplementation likely works by reinforcing a healthy gut microbial community, thereby enhancing the host’s physiological capacity to cope with cold-induced challenges [57]. This includes improved digestion and nutrient absorption, leading to greater energy reserves vital for thermogenesis and maintaining metabolic homeostasis at low temperatures [58]. Furthermore, gut microbiota is known to modulate host immune responses [59]. Probiotics could upregulate key immune genes or pathways, such as the production of antimicrobial peptides (AMPs), which are crucial for fending off secondary infections that may arise during cold stress when the bee’s immune system is compromised [60,61]. Concurrently, the prebiotics likely act synergistically by selectively stimulating the growth and metabolic activity of beneficial endogenous gut bacteria, thereby maximizing the probiotic effect [56]. Our results strongly suggest that these supplements represent a viable strategy for enhancing honey bee resilience in cold climates.

The underlying mechanisms for this enhanced cold resilience are likely multifaceted. Firstly, a healthy gut microbiota, fostered by probiotic and prebiotic intervention, is instrumental in optimizing nutrient digestion and absorption [3]. In cold environments, honey bees exhibit increased metabolic rates to maintain body temperature [62], necessitating efficient energy acquisition. An enhanced gut microbiome can augment the breakdown of complex carbohydrates and facilitate the synthesis of essential vitamins and amino acids, thus providing readily available energy substrates for thermogenesis and metabolic repair [63]. Secondly, probiotics are known to modulate the host’s immune system [64]. Cold stress can suppress immune responses, rendering bees more susceptible to opportunistic pathogens. By enriching beneficial bacterial populations, these supplements may prime the honey bee’s innate immune system, leading to an upregulation of antimicrobial peptides (AMPs) or other immune effectors, thereby bolstering defense against secondary infections commonly associated with cold-induced stress [52,61]. Furthermore, some probiotic strains (*Lactobacillus* and *Bifidobacterium*) possess antioxidant properties, potentially mitigating cold-induced oxidative stress, which contributes to cellular damage and reduced longevity [65]. The observed dose-response relationship implies that a critical threshold or optimal density of beneficial microbes or their metabolites is required to exert these protective effects effectively under cold challenge.

In sharp contrast to cold stress, probiotic and prebiotic supplementation conferred no discernible survival advantage to honey bees exposed to extreme heat (40 °C). All experimental groups, including the control, exhibited a rapid and severe decline in survival, with their respective curves closely converging. This striking lack of efficacy at 40 °C underscores the inherent physiological limits of honey bees and the overwhelming nature of severe thermal shock. This finding suggests that the physiological and cellular damage caused by extreme heat, such as protein denaturation and enzyme dysfunction, overwhelms any potential positive effects of probiotics on gut health or the immune system [66]. The protective mechanisms of probiotics are completely overshadowed by the direct thermal damage. These results are consistent with the findings of Zhao et al. [16], who noted that probiotics may be more effective in enhancing resistance to cold stress (e.g., through improved energy metabolism or immune function under less acute conditions) rather than in combating severe thermal damage. The precipitous drop in survival observed in our 40 °C control group is consistent with the fact that temperatures exceeding the honey bee’s critical thermal maximum become lethal very quickly. At temperatures above 35–36 °C, the metabolic costs for cooling increase dramatically, and sustained temperatures above 38 °C can lead to heat stroke and irreversible cellular damage, including protein denaturation, enzyme inactivation, and membrane disruption [48,59]. Studies on thermal tolerance in various insect species show steep survival curves once temperatures surpass species-specific thresholds [67]. Rachman and Huang [68] specifically highlight heat stress as an emerging threat to honey bees, reviewing how high temperatures directly impact bee physiology and colony health, mirroring the severe mortality rates depicted in our 40 °C survival curve. This underscores that 40 °C represents an acutely lethal condition for individual worker bees, lying completely beyond their physiological compensatory mechanisms.

Regarding the lack of efficacy of supplements under extreme heat stress (40 °C), our results resonate with the general understanding of the severe physiological limitations of honey bees to supra-optimal temperatures. While specific studies directly testing probiotics under such acute, high-temperature stress in bees are less common, the general consensus in insect physiology is that temperatures exceeding a critical thermal maximum lead to rapid and irreversible damage. This damage quickly surpasses the capacity of most internal protective mechanisms, including those potentially enhanced by gut microbiota [48,69]. The immediate denaturing effects of extreme heat on proteins and cellular structures likely overwhelm the more subtle, long-term benefits conferred by gut health. This emphasizes the critical need for behavioral and physical thermoregulation (e.g., fanning, water foraging, hive ventilation, winter isolation, and shading), rather than nutritional supplements, as primary strategies for mitigating severe heat stress in beekeeping practices [70].

Conversely, our results demonstrating high and persistent survival rates in the control group at 35 °C are entirely expected and validate the choice of this temperature as an optimal or “benign” condition for honey bees. This temperature range (32–36 °C) is widely recognized as the optimal brood nest temperature maintained by the colony for successful development and adult bee physiology [71]. At this temperature, metabolic demands for thermoregulation are minimal, and physiological processes operate at peak efficiency, leading to maximum longevity under controlled conditions [62]. The relatively stable survival curve at 35 °C serves as a crucial experimental baseline, emphasizing the importance of thermal homeostasis for honey bee vitality.

Our findings at 4 °C and 15 °C strongly support the view that probiotic and prebiotic supplements act as stress-mitigating agents under cold conditions. These results are consistent with research indicating that a healthy gut microbiota can enhance honey bee metabolism and immune function [50]. While we did not directly analyze the gut microbiota population dynamics, our results are consistent with findings by Liu et al. [50], where microbial health was directly linked to improved physiological and immune responses in honey bees. This further highlights the importance of investigating specific microbiota alteration in future studies.

Cold stress, like any other physiological stressor, can lead to oxidative stress and a rapid depletion of energy reserves [72]. We propose that our supplements help bees preserve vital energy reserves, allowing them to more effectively cope with cold-induced metabolic pressures by improving nutrient absorption and digestive performance. Furthermore, probiotics help strengthen the host immune response [73], which provides a critical advantage under stressful conditions where immune function is compromised [65]. The dose-dependent effect observed in the survival curves potentially suggested that a richer and more stable microbial community, achieved with higher supplement concentrations, provides a greater tier of protection against thermal stresses. These findings align with those of Kwong et al. [59] and Kešnerová et al. [74], who emphasized that a diverse and stable gut microbiome is crucial for maintaining bee health and coping with environmental stressors.

Studies on thermal regulation and heat stress in honey bees have shown that temperatures exceeding the optimal physiological range can be rapidly lethal, regardless of nutritional status, primarily because a bee’s response shifts from active thermoregulation to heat avoidance and basic physiological survival [62]. We showed that nutritional supplementation did not improve the survival of bees, when bees were exposed to a high temperature of 40 °C.

The application of probiotic and prebiotic dietary supplements represents a highly promising strategy to safeguard honey bee health against environmental and physiological stressors. Probiotics, particularly lactic acid bacteria such as *Lactobacillus* and *Bifidobacterium* species, play a fundamental role in maintaining gut microbiota homeostasis, stimulating the host’s immune response, and producing antimicrobial substances that competitively exclude enteric pathogens [28,57]. Furthermore, specific strains of bee-derived lactic acid bacteria have been shown to upregulate host antioxidant defense pathways, mitigating cellular damage induced by excessive oxidative stress [75]. To complement these living microbes, prebiotics (non-digestible carbohydrates such as inulin and fructooligosaccharides) serve as vital metabolic substrates that selectively nourish beneficial endogenous microflora, further enhancing nutrient digestion and gut structural integrity [28,76]. By stabilizing the gut microbiome and dampening oxidative stress, the synergistic use of these dietary compounds offers a powerful mechanism to bolster colony resilience and survival in the face of escalating thermal and environmental challenges. These findings are consistent with our results, which suggest that probiotic and prebiotic food supplements can be promising and beneficial dietary compounds for honey bees. They can help bees overcome gut dysbiosis and increase their resistance to environmental stressors by influencing their immune system and antioxidant defenses. This, in turn, can mitigate the adverse effects of ambient temperature, thus increasing bee survival and longevity [77]. A healthy gut optimizes nutrient absorption and reduces the production of toxic and stressful compounds, which subsequently reduces the oxidative stress burden on the body. Conversely, in the supplemented groups in our study, the presence of probiotics and/or prebiotics acts prophylactically. By reducing the production of stressful compounds (e.g., toxic metabolites, high Reactive Oxygen Species (ROS)), and enhancing gut barrier integrity upstream, overall cellular stress is mitigated. Therefore, the observed reduction in enzymatic activity in the supplement group is not a sign of physiological failure, but rather a beneficial consequence: the body’s defensive system is less challenged, and the high energetic cost of maintaining elevated enzyme activity is spared. The reduction in the oxidative stress burden is achieved through two empirically validated probiotic mechanisms. The first mechanism involves the optimization of the gut environment and metabolism. Probiotics improve digestive system function by producing beneficial metabolites. A healthier, optimized gut ensures more efficient nutrient absorption and systemically reduces toxicity, thereby lowering the overall metabolic and oxidative stress burden imposed on the host cells [78]. The second mechanism is the direct antioxidant contribution of the microflora. Scientific evidence indicates that certain probiotic strains, especially those belonging to the *Lactobacillus* and *Bifidobacterium* genera, possess intrinsic antioxidant properties [79]. These strains can directly contribute to the scavenging of Reactive Oxygen Species (ROS) within the gut lumen, significantly reducing the amount of oxidative damage that would subsequently need to be neutralized by the honey bee’s endogenous cellular enzymes [65]. In summary, the observed reduction in the activity of antioxidant enzymes in the supplemented groups is a clear metric of successful stress mitigation at an earlier stage by the supplements, which is entirely consistent with their established dual role in health modulation and direct antioxidant support.

The findings of this study revealed that at severe cold temperatures (4 °C) compared to moderate cold temperatures (15 °C), the activity of the studied enzymes was impaired and reduced, despite the stress-inducing conditions. This is in agreement with similar results reported in the study by Mucci et al. [65] on SOD and CAT activity. The reason for this change in enzyme activity, as specifically noted in those articles, is the suppression of antioxidant pathways in the cold. Because SOD, CAT, and APX are proteins, extremely high temperatures can lead to their denaturation and a subsequent loss of enzymatic activity. This prevents the enzymes from functioning effectively, even with an increased need for the clearance of reactive oxygen species (ROS). The results of this study regarding survival at high temperatures support this phenomenon and are consistent with the findings in the review article by Rachman and Huang [68]. While we found an increase in enzyme activity at high temperatures (Fig 2), Li et al. [80] showed a short-term heat stress at various temperatures (38, 40, and 42 °C) increased the activity of the SOD, CAT, and POD enzymes in the predatory mite *Neoseiulus barkeri* Hughes*.*

While this study highlights the distinct benefits of synbiotic supplementation, certain limitations remain. The experimental design focused on the combined effect to maximize protection, but did not include separate probiotic and prebiotic groups to isolate individual contributions. Furthermore, this research utilized newly emerged bees to ensure a standardized baseline for assessing the direct efficacy of the supplements. However, we acknowledge that this model bypasses the natural acquisition of gut microbiota through trophallaxis with mature workers, which is a fundamental aspect of bee physiology. Consequently, the absence of an intact, naturally-acquired microbiota as a control group may limit the immediate generalizability of these results to whole-colony dynamics. Additionally, direct gut microbial profiling (e.g., 16S rRNA sequencing) and assessment of antimicrobial peptides (AMPs) were not performed in this study. Future research integrating high-throughput sequencing and transcriptomic analysis of immune genes is needed to fully elucidate the molecular pathways and potential synergistic mechanisms. Finally, field trials will also be essential to validate these laboratory findings in functional apiaries where complex social interactions and natural microbial inoculation occur.

## Conclusions

Giving the multiple negative factors contributing to pollinators decline, understanding and addressing the nutritional requirements of honey bees is critical for maintaining bee colony health. Probiotics, particularly when combined with prebiotics, represent a promising and beneficial dietary supplementation strategy for supporting gut homeostasis and reducing dysbiosis in honey bees. By modulating immune function and antioxidant defenses, these supplements can enhance resilience to environmental stressors, including extreme temperatures. In this study, dietary supplementation was associated with improved survival and physiological stability under adverse thermal conditions. Collectively, these findings highlighted that targeted nutritional interventions may be an effective approach to improving honey bee colonies tolerance to temperature-related stress, thereby supporting colony survival and performance, and apiary management under challenging environmental conditions.

## Supporting information

S1 Fig Plastic bioassay cage specifically designed for testing thermal stress in honey bees. The cage includes two gravity feeder tubes for supplying syrup and water, as well as a side tube for pollen feeding. The cage walls contain ventilation holes to provide airflow.
(PNG)
