# Supplementary figures and images for: Protecting honey bees (Apis mellifera) from thermal stress: Probiotics and prebiotics buffer the survival and antioxidant enzyme activity
Source: PLoS One. 2026 Jul 15;21(7):e0352149. doi: 10.1371/journal.pone.0352149 (PMC13372117; doi:10.1371/journal.pone.0352149)

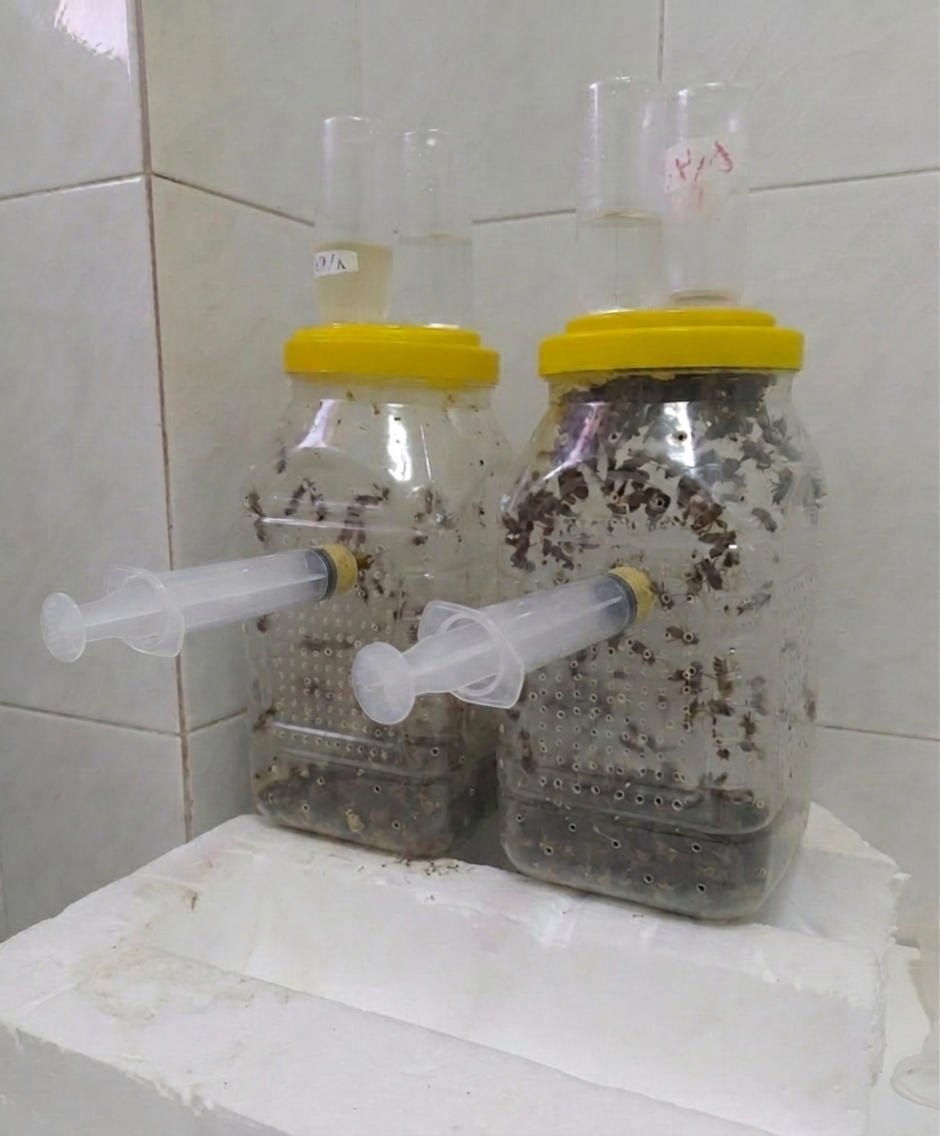

Supplement: S1 Fig — The cage includes two gravity feeder tubes for supplying syrup and water, as well as a side tube for pollen feeding. The cage walls contain ventilation holes to provide airflow. (PNG) [file pone.0352149.s001.png]
