# Supplementary material for: Protecting honey bees (Apis mellifera) from thermal stress: Probiotics and prebiotics buffer the survival and antioxidant enzyme activity
Source: PLoS One. 2026 Jul 15;21(7):e0352149. doi: 10.1371/journal.pone.0352149 (PMC13372117; doi:10.1371/journal.pone.0352149)
Supplement: Attachment — Submitted filename: renamed_45ff8.pdf [file pone.0352149.s002.pdf]

Reviewer's Comments – First Revision

General Comment:

First of all, I would like to congratulate the authors for this study and for their commitment in carrying out this experiment. As a specialist, I am well aware of the challenges involved in handling bees, and I appreciate the effort required. However, several major points require clarification to make the work more scientifically relevant, representative, and robust. In this first revision, I focus on the main concerns. A second revision may address more detailed aspects.

Major Comments:

1. **Rearing Conditions and Experimental Protocol:**
   - The authors report using diets with different concentrations and four temperature levels. It is unclear whether worker bees were reared from emergence (day 0) up to 21 days under these feeding and temperature conditions. Please clarify the exact protocol.
   - The diet provided included syrup and water, but no pollen was given. Since pollen is the main source of protein for bees, its absence raises concerns about the nutritional adequacy of the diet and the interpretation of the results.
   - At what point was mortality recorded – from emergence (day 0) or after 21 days? Why was mortality assessed only after the transfer of bees at day 21? This choice could affect survival outcomes.
   - While the cage is described, including a photograph would improve clarity and reproducibility of the experiment.
   - The rationale for selecting a 21-day period for rearing and measurements should be explained.
2. **Dietary Treatments:**
   - The study applied a combined probiotic + prebiotic treatment. Why were individual treatments (probiotic alone, prebiotic alone) not included? Testing these separately would help determine whether the observed improvements in survival and physiological parameters are due to a synergistic effect or a single component. Although this may be challenging experimentally, it is worth discussing.

3. **Temperature Treatments and Bee Survival:**
    - Regarding the extreme temperature conditions (4°C and 40°C), it is surprising that bees could survive from emergence up to 21 days. Please provide details on the initial number of bees, mortality rates, and sampling losses. Based on experience, high mortality is expected under such conditions, often before 10 days.
4. **Statistical Analysis:**
    - The statistical methods section requires more detail. The software used (e.g., SPSS) should be clearly stated at the beginning of the section.
    - The survival analysis should specify that the Kaplan–Meier estimator was applied.
    - For other parameters, it should be clearly indicated that the factorial design corresponds to a two-way ANOVA, with diet (4 levels) and temperature (4 levels) as factors. This clarification would make the analysis more accurate and understandable.
