# Supplementary material for: Protecting honey bees (Apis mellifera) from thermal stress: Probiotics and prebiotics buffer the survival and antioxidant enzyme activity
Source: PLoS One. 2026 Jul 15;21(7):e0352149. doi: 10.1371/journal.pone.0352149 (PMC13372117; doi:10.1371/journal.pone.0352149)
Supplement: Attachment — Submitted filename: Probiotics and prebiotics. Response to Reviewers.Final.docx [file pone.0352149.s004.docx]

PONE-D-26-03238

Protecting honey bees (Apis mellifera) from thermal stress: Probiotics and prebiotics buffer the survival and antioxidant enzyme activity

PLOS One

**Reviewer #1: Comments for the Authors:**

We would like to express our sincere gratitude for your thorough review of our manuscript. Your insightful comments and constructive feedback have been invaluable in improving the clarity, rigor, and overall quality of this paper. We have carefully considered all your suggestions and addressed each comment in the revised manuscript. All spelling and typographical errors identified have been corrected, and we have performed a comprehensive review of the manuscript to resolve any similar issues. Detailed responses to your specific comments are provided below.

Minor corrections:

- Page 3, line 56: “Howevewr” please, spell it correctly. Done.
- Page 3, line 59: “environmtal” please, spell it correctly. Done.
- Page 3, line 61: “immunoe” please, spell it correctly. Done.
- Page 3, line 62: “bahvious” please, spell it correctly. Done.
- Page 3, line 76: compounds[15], a space is missing. Done.
- Page 4, line 77: “agnist” please, spell it correctly. Done.
- Page 4, line 92: The term “microflora” is no longer preferred. Instead, the community of live microorganisms associated with the host is referred to as “microbiota”. Done.
- Page 5, line 105: “responces”, please, spell it correctly. Done.
- Page 6, line 137: “Treatmnts” please, spell it correctly. The word “include” should be written in the past tense. Done.
- Page 6, line 148: “Temperature stress treatments have been carried out”, please change it for: were carried out. Done.
- Page 22, line 539: “requirments”, please spell it correctly. Done.

Additional comments:

- The authors should consider addressing thermal stress and the importance of temperature maintenance in the Introduction. A brief paragraph would be sufficient. We agree with this suggestion and have added a brief paragraph to the Introduction addressing thermal stress and the importance of temperature maintenance in honey bee colonies.

Lines 140-142: The paragraph “The efficacy of various combinations of Progen and Inulin was evaluated to determine the role of these diets in mitigating the detrimental effects of environmental heat stress on honey bee survival and antioxidant defense system.” should not be included in Material and Methods. We have removed this paragraph from the Materials and Methods section as suggested.

- Lines 145 – 147: “Newly emerged honey bees (1-day-old) were fed a combination of various concentrations of Progen probiotic and Inulin prebiotic over a 21-day period [29]. Following this period, samples were subjected to distinct thermal stress conditions”. For how long were they exposed to the thermal stress? Please, specify this. We have revised the statement to clearly specify the exposure duration.

- Lines 149 – 150: “Each treatment was repeated five times with 100 honey bee individuals (500 honey bees per treatment in total).” It is unclear whether the five repetitions represent independent experiments conducted at different times or simultaneous replicates within the same experiment. Please, clarify. To ensure consistency and minimize experimental error, all five replicates for each treatment were conducted simultaneously. Age-synchronized bees were maintained in separate cages under identical environmental conditions (humidity, photoperiod, and artificial diet) for the 21-day preparatory phase, followed by the thermal stress phase. We have clarified this simultaneous replication in the revised manuscript.

- Line 187: In the “Peroxidase assay”, it is not mentioned how much of honey bee tissue homogenate were added to the reaction mixture. Please, specify it. We extracted 50 µg of protein from 10 µL of hemolymph and added it to the reaction mixture. This detail has now been explicitly stated in the manuscript.

- Figure 1: Please indicate statistically significant differences between treatments in the figure (using asterisks or letters). We have updated Figure 1 to include statistical significance notations. The figure caption has also been updated to define the significance levels used: ns (not significant, *p* > 0.05), (*p* < 0.01), and *** (*p* < 0.0001).

- In the thermal stress results, as well as in the antioxidant activities results, there is repetitive information. The figures are sufficient to explain the results. Any additional comments should be included in the Discussion, not in the Results section. Authors should not discuss in the Results section. We have thoroughly streamlined the Results section. Repetitive information has been deleted, and interpretive or comparative comments have been moved to the Discussion section to maintain a strict separation between results reporting and data interpretation.

- Line 362: The expression “adverse weather conditions” should be revised to “simulated weather conditions,” as the experiments were conducted under laboratory conditions. Done.

- The authors suggest that the gut microbiota was improved and that this explains the observed effects (e.g., increased bee survival). However, no direct analysis of gut microbiota was performed to support this claim. Such conclusions should be revised or rephrased. We completely agree with the reviewer’s important point. Since direct microbial profiling was not conducted in this study, we have revised the manuscript to ensure that our conclusions regarding the gut microbiota are framed as a potential mechanism rather than a definitive finding. We have rephrased the relevant sections to reflect a more cautious interpretation of the survival data.

- Line 477: The study discussed in reference [64] is based on experiments in mice, not in honey bees. I therefore suggest removing this paragraph and restricting the discussion to literature relevant to bees. We agree and have completely removed the paragraph discussing the mice study to keep the discussion strictly focused on bee-relevant literature.

- The authors discuss short-chain fatty acids (SCFAs) and attribute the antioxidant effects observed in the assays to their production. However, SCFAs were not measured in this study. I therefore suggest removing this part of the discussion. Following your suggestion, we have removed all sentences and paragraphs attributing the antioxidant effects directly to SCFA production, as these metabolites were not measured in our assays.

====================================================================================

**Reviewer #2:**

I would like to commend the authors for conducting this study and for their dedication, as handling bees is inherently challenging. However, several key points require clarification to strengthen the scientific relevance and clarity of the work.

We sincerely appreciate your commendable feedback regarding our study. Handling and monitoring honey bees under strict experimental parameters is indeed challenging, and we are grateful for your recognition of our efforts. We have carefully considered each of the key points you raised to strengthen the scientific relevance and clarity of our work. Detailed responses and corresponding manuscript revisions are provided below.

- It is unclear whether worker bees were reared from emergence (day 0) to 21 days under the different diets and temperature conditions. We have revised the Materials and Methods section to clarify that worker bees were indeed reared from emergence (day 0) for 21 days under their respective dietary regimens at optimal temperatures before the thermal stress phase was initiated.
- The timing of mortality assessment should be clarified, as it is not specified whether it was recorded from emergence or only after 21 days. Mortality assessments specifically targeting thermal stress were recorded only after the initial 21-day feeding period. The 21-day period was designed solely to allow for the colonization of probiotic bacteria and the physiological maturation of the young bees. Heat stress treatments were applied immediately following this nutritional phase to evaluate how the established dietary regimens impacted survival against thermal challenges.
- The rationale for choosing a 21-day period for rearing and measurements should also be explained, and including a photograph of the experimental cage would improve reproducibility.

The 21-day period was strategically chosen to cover the critical physiological maturation phase of worker bees (the nursing stage). This duration ensures the full establishment of the gut microbiota and the integration of dietary supplements into the bees' metabolism prior to stress application, aligning with established protocols in honey bee nutritional studies. We have added this rationale to the text. Additionally, a photograph of the experimental cages has been added as **Figure S1 in the Supplementary Material**, and a corresponding reference has been placed in the Materials and Methods section to aid reproducibility.

- Regarding diet composition, the study provided syrup and water but no pollen. Since pollen is the primary protein source for bees, its absence may affect the interpretation of the results. We apologize for omitting this detail in the original draft. In addition to syrup and water, pollen was provided to the bees continuously (*ad libitum*). It was delivered via modified syringes mounted horizontally through the cage walls, allowing the bees direct access to the pollen paste. This critical detail has now been explicitly added to the revised manuscript to prevent any misunderstanding regarding protein availability.
- Furthermore, only a combined probiotic and prebiotic treatment was tested. Testing individual treatments could help determine whether the observed effects are due to a synergistic action or a single component. We agree that testing individual components would yield fascinating comparative data. However, the decision to focus exclusively on the combined application (synbiotics) was based on several strategic and logistical considerations:
  - **Synergistic Rationale:** Prebiotics are specifically utilized as a selective substrate for administered probiotic strains to enhance their colonization and metabolic activity. Our primary objective was to evaluate the maximum potential of this combined nutritional intervention against severe thermal stress, rather than dissecting individual contributions.
  - **Existing Literature:** Previous studies have separately established the baseline benefits of individual probiotics and prebiotics. Our goal was to test the hypothesis that a combined approach provides a more robust physiological buffer.
  - **Experimental Scale:** Including individual treatment groups for every concentration across four temperature regimes would have tripled our experimental units, making daily monitoring and biochemical sampling logistically unfeasible for this specific study. As suggested, we have added a paragraph in the Discussion section formally acknowledging this limitation.
- Concerning temperature treatments, the reported survival under extreme conditions of 4°C and 40°C from day 0 to 21 seems unlikely. Details on the initial number of bees, mortality rates, and any sampling losses should be provided to justify survival under these conditions. We realize our initial description of the experimental timeline caused confusion, and we completely agree that survival under those extremes for 21 days would be highly unlikely. To clarify:
  - **Pre-treatment Period (Day 1 to 21):** Newly emerged bees were maintained under optimal laboratory conditions (35°C and 60% RH) for 21 days. No thermal stress was applied during this phase. Natural mortality was negligible.
  - **Thermal Stress Phase (After Day 21):** The extreme thermal stresses (4, 15, and 40°C) were applied *only after* this 21-day pre-treatment period.

**Survival Observations under Stress:** Once thermal stress began, mortality rates increased rapidly, exactly as expected. At 40°C, 100% mortality was reached within approximately 4 days. We have revised the Abstract and Materials & Methods sections to clearly distinguish between the benign pre-treatment phase and the acute thermal stress phase.

- The statistical methods section also requires clarification. The software used, such as SPSS, should be stated at the beginning of the analysis section. Survival analysis should specify that the Kaplan–Meier estimator was applied, while other parameters should be analyzed using a two-way ANOVA with diet and temperature as the two factors. The Statistical Methods section has been completely revised based on your recommendations. We have moved the software information (SPSS) to the beginning of the section. We explicitly state the use of the Kaplan–Meier estimator for survival analysis. Furthermore, we have clarified that a two-way ANOVA (factorial design) was employed for all other parameters, specifying diet and temperature as the primary factors.
- In summary, this study is valuable and the work is commendable, but clarification on the rearing protocol, diet, temperature conditions, and statistical analysis is essential to strengthen the manuscript, improve reproducibility, and increase its scientific impact. Thank you.

===================================================================================

Reviewer #3:

This study is very timely in the era of global climate change, as winters are becoming increasingly harsh and honey bee health is declining due to cold-season nutritional deficiencies. Supplementation with pre- and probiotics is therefore important. The study is well written and suitable for minor revision.

We sincerely thank you for this constructive critique and for recognizing the timeliness and relevance of this study in the context of global climate change. We have carefully considered each of your points and incorporated your recommended structural and conceptual changes into the revised manuscript. Detailed responses are provided below.

1. Lines 63–64: The claim that stress leads to bee population losses is overstated. Stressors can threaten colony health, but they do not necessarily cause population decline, particularly in managed honey bees, where resilience, husbandry practices, and nutrition mitigate impacts. Please temper this statement and, throughout the manuscript, avoid overgeneralizations by distinguishing colony-level stress from population-level trends. Support any population-level claims with appropriate, recent citations and revise the wording to reflect the current evidence base. As suggested, we have tempered our statements regarding population-level declines. We revised the manuscript to clearly distinguish between localized colony-level stress and broader population trends, explicitly acknowledging that managed bees often exhibit high resilience due to modern husbandry practices and supplemental nutrition. We have also added recent citations to support these nuanced points.

2. Hemagglutinins- check spelling it is the same spelling in the google. Done.

3. Line 68: Please insert a paragraph break to separate the oxidative stress discussion from the preceding text, improving readability and logical flow. Done.

4. In the final paragraph, please add a dedicated, well-structured section on honeybee–thermal–microbiome interactions. Summarize how the gut microbiome responds under thermal stress (both heat waves and cold spells), and how these shifts may influence resilience, colony health, and pathogen susceptibility. Highlight mechanisms by which bees maintain their microbiome and how the microbiome may, in turn, bolster bee resilience. After this synthesis, transition to probiotics and prebiotics, explaining how they may directly or indirectly support the gut microbiome. To create a clear, “funnel-shaped” narrative: start with thermal stress impacts, then discuss bee–microbiome resilience mechanisms, and conclude with interventions (probiotics/prebiotics). Please incorporate and appropriately cite relevant studies, for example:

• High temperatures: “Hot and Bothered: Bees’ Gut Microbiome Shifts Under Thermal Stress and Pathogen Infection” (J.I. Van Wyk et al., 2025); “Thermal niches of specialized gut symbionts: the case of social bees” (T.J. Hammer, E. Le, N.A. Moran).
• Low temperatures: “High abundance of lactobacilli in the gut microbiome of honey bees during winter” (Brar et al., 2025); “Overwintering Honey Bee Colonies: Effect of Worker Age and Climate on the Hindgut Microbiota” (P.W. Maes, 2021); “Gut microbiota structure differs between honeybees in winter and summer” (L. Kešnerová, 2020). Following your excellent suggestion, we have added a well-structured section on honey bee–thermal–microbiome interactions to the Introduction. Utilizing the recommended 'funnel-shaped' narrative, we synthesized how extreme heat and cold stress disrupt the gut microbiome (incorporating the suggested citations by Van Wyk et al., Brar et al., etc.) and how these shifts influence colony health. We used this synthesis to firmly justify the use of prebiotics and probiotics as a strategic intervention to bolster resilience against climatic extremes.

5. Material and methods: Please specify the exact months that constitute the summer and autumn seasons for 2023–2024 in your study. We sincerely thank you for catching this discrepancy. This was a translation and drafting oversight in the original manuscript. The study was actually conducted during the spring and summer seasons, specifically from March 2024 to September 2024. We have corrected these dates and specified the exact months in the revised manuscript.

6. Paragraph 2: Please include a clear photograph/figure of your experimental setup to improve readability and understanding. Add this image to the supplementary materials. We have added a clear photograph of our cage setup, complete with feeding and water systems, as **Figure S1** in the Supplementary Materials.

7. Commercial probiotic Progen and prebiotic inulin: please specify their composition. This is very important. We have updated the “Preparation of dietary supplements” section to provide the specific microbial composition of the Progen® probiotic (a multi-strain formulation) and the chemical nature of the inulin used (a long-chain fructooligosaccharide derived from chicory root).

8. Please clarify the timeline: “Following this period” is ambiguous. For how many days was the reference control maintained? Was feeding conducted for 21 days and then samples moved to thermal treatment, or did these occur concurrently? The current wording is confusing; please specify the exact durations and sequence for each group. We have clarified the timeline and revised the statement.

9. Newly emerged bees do not have an intact gut microbiota because most bacterial species are acquired through trophallaxis, a key component of bee physiology. Bees with an intact microbiota behave and perform physiologically better than newly emerged bees lacking it. Why did the authors not include a treatment with bees having an intact microbiota? For example, newly emerged bees could be marked and co-housed with mature workers to inoculate their microbiome, serving as a control group. In practical applications, probiotics are fed to whole colonies comprising workers of different ages and castes, not only to newly emerged bees. Please provide a clear justification for the chosen treatments and controls and explain why a control group with an intact microbiota was not included. You raise an excellent point regarding the natural acquisition of microbiota through trophallaxis, a fundamental biological reality of hive dynamics. We acknowledge that using isolated newly emerged bees provides a highly 'controlled' view that does not encapsulate the complexity of a mature colony's social environment. Cohousing newly emerged bees with mature workers for natural inoculation would indeed serve as an ideal control group. While executing this was outside the scope of our current laboratory setup, we have explicitly addressed this limitation in the final paragraph of the Discussion section. We are committed to including a 'natural microbiota' treatment group in our future field studies to bridge the gap between laboratory bioassays and real-world colony applications.

10. Lines 338–340: This is an interesting observation. Please discuss how these temperatures may affect the gut microbiota, particularly lactobacilli (Lactobacillus spp.), which are often highly abundant under such conditions. Since direct microbial profiling was not conducted in this specific study, we have carefully rephrased our conclusions regarding gut microbiota shifts to frame them as potential mechanisms rather than definitive findings.

11. Line 366: Please refer back to the suggested citations and include them in this section as appropriate. We have successfully integrated the suggested citations into this section of the Discussion to strengthen the context.

12. Please add a dedicated paragraph on the study’s limitations (e.g., the absence of gut microbial analysis and limited assessment of antimicrobial peptides), and outline future studies that could be conducted based on these data to strengthen the conclusions. We have added a dedicated paragraph at the end of the Discussion section. In it, we explicitly state the lack of direct gut microbial analysis and the exclusion of antimicrobial peptide (AMP) assessments as limitations. Furthermore, we outline how future research utilizing 16S rRNA sequencing and transcriptomic analysis of immune genes will build upon these findings to provide a comprehensive understanding of synbiotic-mediated stress resilience.
