# Supplementary material for: Protecting honey bees (Apis mellifera) from thermal stress: Probiotics and prebiotics buffer the survival and antioxidant enzyme activity
Source: PLoS One. 2026 Jul 15;21(7):e0352149. doi: 10.1371/journal.pone.0352149 (PMC13372117; doi:10.1371/journal.pone.0352149)
Supplement: Peer review history

# Peer review: Decision Letter 0

**DOI:** 10.1371/journal.pone.0352149.r001

**Authors:** Vicente Martínez López

18 Apr 2026

PONE-D-26-03238

Protecting honey bees (Apis mellifera) from thermal stress: Probiotics and prebiotics buffer the survival and antioxidant enzyme activity

PLOS One

Dear Dr. Bahreini,

Thank you for submitting your manuscript to PLOS ONE. After careful consideration, we feel that it has merit but does not fully meet PLOS ONE’s publication criteria as it currently stands. Therefore, we invite you to submit a revised version of the manuscript that addresses the points raised during the review process.

Please submit your revised manuscript by Jun 02 2026 11:59PM. If you will need more time than this to complete your revisions, please reply to this message or contact the journal office at plosone@plos.org. When you're ready to submit your revision, log on to https://www.editorialmanager.com/pone/ and select the 'Submissions Needing Revision' folder to locate your manuscript file.

Please include the following items when submitting your revised manuscript:

- A letter that responds to each point raised by the academic editor and reviewer(s). You should upload this letter as a separate file labeled 'Response to Reviewers'.
- A marked-up copy of your manuscript that highlights changes made to the original version. You should upload this as a separate file labeled 'Revised Manuscript with Track Changes'.
- An unmarked version of your revised paper without tracked changes. You should upload this as a separate file labeled 'Manuscript'.

If you would like to make changes to your financial disclosure, please include your updated statement in your cover letter. Guidelines for resubmitting your figure files are available below the reviewer comments at the end of this letter.

If applicable, we recommend that you deposit your laboratory protocols in protocols.io to enhance the reproducibility of your results. Protocols.io assigns your protocol its own identifier (DOI) so that it can be cited independently in the future. For instructions see: https://journals.plos.org/plosone/s/submission-guidelines#loc-laboratory-protocols. Additionally, PLOS ONE offers an option for publishing peer-reviewed Lab Protocol articles, which describe protocols hosted on protocols.io. Read more information on sharing protocols at https://plos.org/protocols?utm_medium=editorial-email&utm_source=authorletters&utm_campaign=protocols.

As the corresponding author, your ORCID iD is verified in the submission system and will appear in the published article. PLOS supports the use of ORCID, and we encourage all coauthors to register for an ORCID iD and use it as well. Please encourage your coauthors to verify their ORCID iD within the submission system before final acceptance, as unverified ORCID iDs will not appear in the published article. *Only*  the individual author can complete the verification step; PLOS staff *cannot*  verify ORCID iDs on behalf of authors.

We look forward to receiving your revised manuscript.

Kind regards,

Vicente Martínez López

Academic Editor

PLOS One

Journal Requirements:

When submitting your revision, we need you to address these additional requirements.

1.Please ensure that your manuscript meets PLOS ONE's style requirements, including those for file naming. The PLOS ONE style templates can be found at

https://journals.plos.org/plosone/s/file?id=wjVg/PLOSOne_formatting_sample_main_body.pdf and

https://journals.plos.org/plosone/s/file?id=ba62/PLOSOne_formatting_sample_title_authors_affiliations.pdf

2. We note that the grant information you provided in the ‘Funding Information’ and ‘Financial Disclosure’ sections do not match.

When you resubmit, please ensure that you provide the correct grant numbers for the awards you received for your study in the ‘Funding Information’ section.

3. Thank you for stating the following financial disclosure:

“University of Zabol”

Please state what role the funders took in the study.  If the funders had no role, please state: "The funders had no role in study design, data collection and analysis, decision to publish, or preparation of the manuscript."

If this statement is not correct you must amend it as needed.

Please include this amended Role of Funder statement in your cover letter; we will change the online submission form on your behalf.

4. We notice that your supplementary figures are uploaded with the file type 'Figure'. Please amend the file type to 'Supporting Information'. Please ensure that each Supporting Information file has a legend listed in the manuscript after the references list.

5. We note that your Data Availability Statement is currently as follows: “All relevant data are within the manuscript and its Supporting Information files.”

Please confirm at this time whether or not your submission contains all raw data required to replicate the results of your study. Authors must share the “minimal data set” for their submission. PLOS defines the minimal data set to consist of the data required to replicate all study findings reported in the article, as well as related metadata and methods (https://journals.plos.org/plosone/s/data-availability#loc-minimal-data-set-definition).

For example, authors should submit the following data:

- The values behind the means, standard deviations and other measures reported;

- The values used to build graphs;

- The points extracted from images for analysis

Authors do not need to submit their entire data set if only a portion of the data was used in the reported study.

If your submission does not contain these data, please either upload them as Supporting Information files or deposit them to a stable, public repository and provide us with the relevant URLs, DOIs, or accession numbers. For a list of recommended repositories, please see https://journals.plos.org/plosone/s/recommended-repositories.

If there are ethical or legal restrictions on sharing a de-identified data set, please explain them in detail (e.g., data contain potentially sensitive information, data are owned by a third-party organization, etc.) and who has imposed them (e.g., an ethics committee). Please also provide contact information for a data access committee, ethics committee, or other institutional body to which data requests may be sent. If data are owned by a third party, please indicate how others may request data access.

6. If the reviewer comments include a recommendation to cite specific previously published works, please review and evaluate these publications to determine whether they are relevant and should be cited. There is no requirement to cite these works unless the editor has indicated otherwise.

Additional Editor Comments:

The three reviewers acknowledge the value of this study and commend the authors for their work. However, they have also identified several issues that need to be addressed before the manuscript can be considered for publication. Minor typographical errors should be corrected throughout the manuscript. More importantly, several aspects of the methodology require further clarification, including the rearing conditions, experimental protocol and dietary treatments. In addition, the statistical analyses need to be described in greater detail to ensure clarity and reproducibility.

The reviewers have also provided useful suggestions to strengthen both the Introduction and the Discussion sections, which should be carefully considered. I encourage the authors to address all of these points thoroughly. I would be pleased to reconsider the manuscript for publication in *PLOS ONE*  once a revised version has been submitted.

[Note: HTML markup is below. Please do not edit.]

Reviewer's Responses to Questions

**Comments to the Author**

1. Is the manuscript technically sound, and do the data support the conclusions?

The manuscript must describe a technically sound piece of scientific research with data that supports the conclusions. Experiments must have been conducted rigorously, with appropriate controls, replication, and sample sizes. The conclusions must be drawn appropriately based on the data presented.

Reviewer #1: Yes

Reviewer #2: Partly

Reviewer #3: Yes

**********

2. Has the statistical analysis been performed appropriately and rigorously?

Reviewer #1: Yes

Reviewer #2: Yes

Reviewer #3: Yes

**********

3. Have the authors made all data underlying the findings in their manuscript fully available?

The PLOS Data policy requires authors to make all data underlying the findings described in their manuscript fully available without restriction, with rare exception (please refer to the Data Availability Statement in the manuscript PDF file). The data should be provided as part of the manuscript or its supporting information, or deposited to a public repository. For example, in addition to summary statistics, the data points behind means, medians and variance measures should be available. If there are restrictions on publicly sharing data—e.g. participant privacy or use of data from a third party—those must be specified.

Reviewer #1: Yes

Reviewer #2: Yes

Reviewer #3: Yes

**********

4. Is the manuscript presented in an intelligible fashion and written in standard English?

PLOS ONE does not copyedit accepted manuscripts, so the language in submitted articles must be clear, correct, and unambiguous. Any typographical or grammatical errors should be corrected at revision, so please note any specific errors here.

Reviewer #1: Yes

Reviewer #2: No

Reviewer #3: Yes

**********

5. Review Comments to the Author

Please use the space provided to explain your answers to the questions above. You may also include additional comments for the author, including concerns about dual publication, research ethics, or publication ethics. (Please upload your review as an attachment if it exceeds 20,000 characters)

Reviewer #1: Comments for the Authors:

Minor corrections:

- Page 3, line 56: “Howevewr” please, spell it correctly.

- Page 3, line 59: “environmtal” please, spell it correctly.

- Page 3, line 61: “immunoe” please, spell it correctly.

- Page 3, line 62: “bahvious” please, spell it correctly.

- Page 3, line 76: compounds[15], a space is missing.

- Page 4, line 77: “agnist” please, spell it correctly.

- Page 4, line 92: The term “microflora” is no longer preferred. Instead, the community of live microorganisms associated with the host is referred to as “microbiota”.

- Page 5, line 105: “responces”, please, spell it correctly.

- Page 6, line 137: “Treatmnts” please, spell it correctly. The word “include” should be written in the past tense.

- Page 6, line 148: “Temperature stress treatments have been carried out”, please change it for: were carried out.

- Page 22, line 539: “requirments”, please spell it correctly.

Additional comments:

- The authors should consider addressing thermal stress and the importance of temperature maintenance in the Introduction. A brief paragraph would be sufficient.

- Lines 140-142: The paragraph “The efficacy of various combinations of Progen and Inulin was evaluated to determine the role of these diets in mitigating the detrimental effects of environmental heat stress on honey bee survival and antioxidant defense system.” should not be included in Material and Methods.

- Lines 145 – 147: “Newly emerged honey bees (1-day-old) were fed a combination of various concentrations of Progen probiotic and Inulin prebiotic over a 21-day period [29]. Following this period, samples were subjected to distinct thermal stress conditions”. For how long were they exposed to the thermal stress? Please, specify this.

- Lines 149 – 150: “Each treatment was repeated five times with 100 honey bee individuals (500 honey bees per treatment in total).” It is unclear whether the five repetitions represent independent experiments conducted at different times or simultaneous replicates within the same experiment. Please, clarify.

- Line 187: In the “Peroxidase assay”, it is not mentioned how much of honey bee tissue homogenate were added to the reaction mixture. Please, specify it.

- Figure 1: Please indicate statistically significant differences between treatments in the figure (using asterisks or letters).

- In the thermal stress results, as well as in the antioxidant activities results, there is repetitive information. The figures are sufficient to explain the results. Any additional comments should be included in the Discussion, not in the Results section. Authors should not discuss in the Results section.

- Line 362: The expression “adverse weather conditions” should be revised to “simulated weather conditions,” as the experiments were conducted under laboratory conditions.

- The authors suggest that the gut microbiota was improved and that this explains the observed effects (e.g., increased bee survival). However, no direct analysis of gut microbiota was performed to support this claim. Such conclusions should be revised or rephrased.

- Line 477: The study discussed in reference [64] is based on experiments in mice, not in honey bees. I therefore suggest removing this paragraph and restricting the discussion to literature relevant to bees.

- The authors discuss short-chain fatty acids (SCFAs) and attribute the antioxidant effects observed in the assays to their production. However, SCFAs were not measured in this study. I therefore suggest removing this part of the discussion.

Reviewer #2: I would like to commend the authors for conducting this study and for their dedication, as handling bees is inherently challenging. However, several key points require clarification to strengthen the scientific relevance and clarity of the work. It is unclear whether worker bees were reared from emergence (day 0) to 21 days under the different diets and temperature conditions. The timing of mortality assessment should be clarified, as it is not specified whether it was recorded from emergence or only after 21 days. The rationale for choosing a 21-day period for rearing and measurements should also be explained, and including a photograph of the experimental cage would improve reproducibility. Regarding diet composition, the study provided syrup and water but no pollen. Since pollen is the primary protein source for bees, its absence may affect the interpretation of the results. Furthermore, only a combined probiotic and prebiotic treatment was tested. Testing individual treatments could help determine whether the observed effects are due to a synergistic action or a single component. Concerning temperature treatments, the reported survival under extreme conditions of 4°C and 40°C from day 0 to 21 seems unlikely. Details on the initial number of bees, mortality rates, and any sampling losses should be provided to justify survival under these conditions. The statistical methods section also requires clarification. The software used, such as SPSS, should be stated at the beginning of the analysis section. Survival analysis should specify that the Kaplan–Meier estimator was applied, while other parameters should be analyzed using a two-way ANOVA with diet and temperature as the two factors. In summary, this study is valuable and the work is commendable, but clarification on the rearing protocol, diet, temperature conditions, and statistical analysis is essential to strengthen the manuscript, improve reproducibility, and increase its scientific impact.

Reviewer #3: This study is very timely in the era of global climate change, as winters are becoming increasingly harsh and honey bee health is declining due to cold-season nutritional deficiencies. Supplementation with pre- and probiotics is therefore important. The study is well written and suitable for minor revision.

1. Lines 63–64: The claim that stress leads to bee population losses is overstated. Stressors can threaten colony health, but they do not necessarily cause population decline, particularly in managed honey bees, where resilience, husbandry practices, and nutrition mitigate impacts. Please temper this statement and, throughout the manuscript, avoid overgeneralizations by distinguishing colony-level stress from population-level trends. Support any population-level claims with appropriate, recent citations and revise the wording to reflect the current evidence base.

2. Hemagglutinins- check spelling

3. Line 68: Please insert a paragraph break to separate the oxidative stress discussion from the preceding text, improving readability and logical flow.

4. In the final paragraph, please add a dedicated, well-structured section on honeybee–thermal–microbiome interactions. Summarize how the gut microbiome responds under thermal stress (both heat waves and cold spells), and how these shifts may influence resilience, colony health, and pathogen susceptibility. Highlight mechanisms by which bees maintain their microbiome and how the microbiome may, in turn, bolster bee resilience. After this synthesis, transition to probiotics and prebiotics, explaining how they may directly or indirectly support the gut microbiome. To create a clear, “funnel-shaped” narrative: start with thermal stress impacts, then discuss bee–microbiome resilience mechanisms, and conclude with interventions (probiotics/prebiotics). Please incorporate and appropriately cite relevant studies, for example:

• High temperatures: “Hot and Bothered: Bees’ Gut Microbiome Shifts Under Thermal Stress and Pathogen Infection” (J.I. Van Wyk et al., 2025); “Thermal niches of specialized gut symbionts: the case of social bees” (T.J. Hammer, E. Le, N.A. Moran).

• Low temperatures: “High abundance of lactobacilli in the gut microbiome of honey bees during winter” (Brar et al., 2025); “Overwintering Honey Bee Colonies: Effect of Worker Age and Climate on the Hindgut Microbiota” (P.W. Maes, 2021); “Gut microbiota structure differs between honeybees in winter and summer” (L. Kešnerová, 2020)

5. Material and methods: Please specify the exact months that constitute the summer and autumn seasons for 2023–2024 in your study.

6. Paragraph 2: Please include a clear photograph/figure of your experimental setup to improve readability and understanding. Add this image to the supplementary materials.

7. Commercial probiotic Progen and prebiotic inulin: please specify their composition. This is very important.

8. Please clarify the timeline: “Following this period” is ambiguous. For how many days was the reference control maintained? Was feeding conducted for 21 days and then samples moved to thermal treatment, or did these occur concurrently? The current wording is confusing; please specify the exact durations and sequence for each group.

9. Newly emerged bees do not have an intact gut microbiota because most bacterial species are acquired through trophallaxis, a key component of bee physiology. Bees with an intact microbiota behave and perform physiologically better than newly emerged bees lacking it. Why did the authors not include a treatment with bees having an intact microbiota? For example, newly emerged bees could be marked and co-housed with mature workers to inoculate their microbiome, serving as a control group. In practical applications, probiotics are fed to whole colonies comprising workers of different ages and castes, not only to newly emerged bees. Please provide a clear justification for the chosen treatments and controls and explain why a control group with an intact microbiota was not included.

10. Lines 338–340: This is an interesting observation. Please discuss how these temperatures may affect the gut microbiota, particularly lactobacilli (Lactobacillus spp.), which are often highly abundant under such conditions.

11. Line 366: Please refer back to the suggested citations and include them in this section as appropriate.

12. Please add a dedicated paragraph on the study’s limitations (e.g., the absence of gut microbial analysis and limited assessment of antimicrobial peptides), and outline future studies that could be conducted based on these data to strengthen the conclusions.

**********

6. PLOS authors have the option to publish the peer review history of their article (what does this mean?). If published, this will include your full peer review and any attached files.

If you choose “no”, your identity will remain anonymous but your review may still be made public.

**Do you want your identity to be public for this peer review?**  For information about this choice, including consent withdrawal, please see our Privacy Policy.

Reviewer #1: No

Reviewer #2: No

Reviewer #3: No

**********

[NOTE: If reviewer comments were submitted as an attachment file, they will be attached to this email and accessible via the submission site. Please log into your account, locate the manuscript record, and check for the action link "View Attachments". If this link does not appear, there are no attachment files.]

To ensure your figures meet our technical requirements, please review our figure guidelines: https://journals.plos.org/plosone/s/figures

You may also use PLOS’s free figure tool, NAAS, to help you prepare publication quality figures: https://journals.plos.org/plosone/s/figures#loc-tools-for-figure-preparation.

NAAS will assess whether your figures meet our technical requirements by comparing each figure against our figure specifications.

Attachment Submitted filename: renamed_45ff8.pdf

# Author comment: Author response to Decision Letter 1

**DOI:** 10.1371/journal.pone.0352149.r002

28 May 2026

Reviewer #1: Comments for the Authors:

We would like to express our sincere gratitude for your thorough review of our manuscript. Your insightful comments and constructive feedback have been invaluable in improving the clarity, rigor, and overall quality of this paper. We have carefully considered all your suggestions and addressed each comment in the revised manuscript. All spelling and typographical errors identified have been corrected, and we have performed a comprehensive review of the manuscript to resolve any similar issues. Detailed responses to your specific comments are provided below.

Minor corrections:

- Page 3, line 56: “Howevewr” please, spell it correctly. Done.

- Page 3, line 59: “environmtal” please, spell it correctly. Done.

- Page 3, line 61: “immunoe” please, spell it correctly. Done.

- Page 3, line 62: “bahvious” please, spell it correctly. Done.

- Page 3, line 76: compounds[15], a space is missing. Done.

- Page 4, line 77: “agnist” please, spell it correctly. Done.

- Page 4, line 92: The term “microflora” is no longer preferred. Instead, the community of live microorganisms associated with the host is referred to as “microbiota”. Done.

- Page 5, line 105: “responces”, please, spell it correctly. Done.

- Page 6, line 137: “Treatmnts” please, spell it correctly. The word “include” should be written in the past tense. Done.

- Page 6, line 148: “Temperature stress treatments have been carried out”, please change it for: were carried out. Done.

- Page 22, line 539: “requirments”, please spell it correctly. Done.

Additional comments:

- The authors should consider addressing thermal stress and the importance of temperature maintenance in the Introduction. A brief paragraph would be sufficient. We agree with this suggestion and have added a brief paragraph to the Introduction addressing thermal stress and the importance of temperature maintenance in honey bee colonies.

Lines 140-142: The paragraph “The efficacy of various combinations of Progen and Inulin was evaluated to determine the role of these diets in mitigating the detrimental effects of environmental heat stress on honey bee survival and antioxidant defense system.” should not be included in Material and Methods. We have removed this paragraph from the Materials and Methods section as suggested.

- Lines 145 – 147: “Newly emerged honey bees (1-day-old) were fed a combination of various concentrations of Progen probiotic and Inulin prebiotic over a 21-day period [29]. Following this period, samples were subjected to distinct thermal stress conditions”. For how long were they exposed to the thermal stress? Please, specify this. We have revised the statement to clearly specify the exposure duration.

- Lines 149 – 150: “Each treatment was repeated five times with 100 honey bee individuals (500 honey bees per treatment in total).” It is unclear whether the five repetitions represent independent experiments conducted at different times or simultaneous replicates within the same experiment. Please, clarify. To ensure consistency and minimize experimental error, all five replicates for each treatment were conducted simultaneously. Age-synchronized bees were maintained in separate cages under identical environmental conditions (humidity, photoperiod, and artificial diet) for the 21-day preparatory phase, followed by the thermal stress phase. We have clarified this simultaneous replication in the revised manuscript.

- Line 187: In the “Peroxidase assay”, it is not mentioned how much of honey bee tissue homogenate were added to the reaction mixture. Please, specify it. We extracted 50 µg of protein from 10 µL of hemolymph and added it to the reaction mixture. This detail has now been explicitly stated in the manuscript.

- Figure 1: Please indicate statistically significant differences between treatments in the figure (using asterisks or letters). We have updated Figure 1 to include statistical significance notations. The figure caption has also been updated to define the significance levels used: ns (not significant, p > 0.05), (p < 0.01), and *** (p < 0.0001).

- In the thermal stress results, as well as in the antioxidant activities results, there is repetitive information. The figures are sufficient to explain the results. Any additional comments should be included in the Discussion, not in the Results section. Authors should not discuss in the Results section. We have thoroughly streamlined the Results section. Repetitive information has been deleted, and interpretive or comparative comments have been moved to the Discussion section to maintain a strict separation between results reporting and data interpretation.

- Line 362: The expression “adverse weather conditions” should be revised to “simulated weather conditions,” as the experiments were conducted under laboratory conditions. Done.

- The authors suggest that the gut microbiota was improved and that this explains the observed effects (e.g., increased bee survival). However, no direct analysis of gut microbiota was performed to support this claim. Such conclusions should be revised or rephrased. We completely agree with the reviewer’s important point. Since direct microbial profiling was not conducted in this study, we have revised the manuscript to ensure that our conclusions regarding the gut microbiota are framed as a potential mechanism rather than a definitive finding. We have rephrased the relevant sections to reflect a more cautious interpretation of the survival data.

- Line 477: The study discussed in reference [64] is based on experiments in mice, not in honey bees. I therefore suggest removing this paragraph and restricting the discussion to literature relevant to bees. We agree and have completely removed the paragraph discussing the mice study to keep the discussion strictly focused on bee-relevant literature.

- The authors discuss short-chain fatty acids (SCFAs) and attribute the antioxidant effects observed in the assays to their production. However, SCFAs were not measured in this study. I therefore suggest removing this part of the discussion. Following your suggestion, we have removed all sentences and paragraphs attributing the antioxidant effects directly to SCFA production, as these metabolites were not measured in our assays.

====================================================================================

Reviewer #2:

I would like to commend the authors for conducting this study and for their dedication, as handling bees is inherently challenging. However, several key points require clarification to strengthen the scientific relevance and clarity of the work.

We sincerely appreciate your commendable feedback regarding our study. Handling and monitoring honey bees under strict experimental parameters is indeed challenging, and we are grateful for your recognition of our efforts. We have carefully considered each of the key points you raised to strengthen the scientific relevance and clarity of our work. Detailed responses and corresponding manuscript revisions are provided below.

• It is unclear whether worker bees were reared from emergence (day 0) to 21 days under the different diets and temperature conditions. We have revised the Materials and Methods section to clarify that worker bees were indeed reared from emergence (day 0) for 21 days under their respective dietary regimens at optimal temperatures before the thermal stress phase was initiated.

• The timing of mortality assessment should be clarified, as it is not specified whether it was recorded from emergence or only after 21 days. Mortality assessments specifically targeting thermal stress were recorded only after the initial 21-day feeding period. The 21-day period was designed solely to allow for the colonization of probiotic bacteria and the physiological maturation of the young bees. Heat stress treatments were applied immediately following this nutritional phase to evaluate how the established dietary regimens impacted survival against thermal challenges.

• The rationale for choosing a 21-day period for rearing and measurements should also be explained, and including a photograph of the experimental cage would improve reproducibility.

The 21-day period was strategically chosen to cover the critical physiological maturation phase of worker bees (the nursing stage). This duration ensures the full establishment of the gut microbiota and the integration of dietary supplements into the bees' metabolism prior to stress application, aligning with established protocols in honey bee nutritional studies. We have added this rationale to the text. Additionally, a photograph of the experimental cages has been added as Figure S1 in the Supplementary Material, and a corresponding reference has been placed in the Materials and Methods section to aid reproducibility.

• Regarding diet composition, the study provided syrup and water but no pollen. Since pollen is the primary protein source for bees, its absence may affect the interpretation of the results. We apologize for omitting this detail in the original draft. In addition to syrup and water, pollen was provided to the bees continuously (ad libitum). It was delivered via modified syringes mounted horizontally through the cage walls, allowing the bees direct access to the pollen paste. This critical detail has now been explicitly added to the revised manuscript to prevent any misunderstanding regarding protein availability.

• Furthermore, only a combined probiotic and prebiotic treatment was tested. Testing individual treatments could help determine whether the observed effects are due to a synergistic action or a single component. We agree that testing individual components would yield fascinating comparative data. However, the decision to focus exclusively on the combined application (synbiotics) was based on several strategic and logistical considerations:

o Synergistic Rationale: Prebiotics are specifically utilized as a selective substrate for administered probiotic strains to enhance their colonization and metabolic activity. Our primary objective was to evaluate the maximum potential of this combined nutritional intervention against severe thermal stress, rather than dissecting individual contributions.

o Existing Literature: Previous studies have separately established the baseline benefits of individual probiotics and prebiotics. Our goal was to test the hypothesis that a combined approach provides a more robust physiological buffer.

o Experimental Scale: Including individual treatment groups for every concentration across four temperature regimes would have tripled our experimental units, making daily monitoring and biochemical sampling logistically unfeasible for this specific study. As suggested, we have added a paragraph in the Discussion section formally acknowledging this limitation.

• Concerning temperature treatments, the reported survival under extreme conditions of 4°C and 40°C from day 0 to 21 seems unlikely. Details on the initial number of bees, mortality rates, and any sampling losses should be provided to justify survival under these conditions. We realize our initial description of the experimental timeline caused confusion, and we completely agree that survival under those extremes for 21 days would be highly unlikely. To clarify:

o Pre-treatment Period (Day 1 to 21): Newly emerged bees were maintained under optimal laboratory conditions (35°C and 60% RH) for 21 days. No thermal stress was applied during this phase. Natural mortality was negligible.

o Thermal Stress Phase (After Day 21): The extreme thermal stresses (4, 15, and 40°C) were applied only after this 21-day pre-treatment period.

Survival Observations under Stress: Once thermal stress began, mortality rates increased rapidly, exactly as expected. At 40°C, 100% mortality was reached within approximately 4 days. We have revised the Abstract and Materials & Methods sections to clearly distinguish between the benign pre-treatment phase and the acute thermal stress phase.

• The statistical methods section also requires clarification. The software used, such as SPSS, should be stated at the beginning of the analysis section. Survival analysis should specify that the Kaplan–Meier estimator was applied, while other parameters should be analyzed using a two-way ANOVA with diet and temperature as the two factors. The Statistical Methods section has been completely revised based on your recommendations. We have moved the software information (SPSS) to the beginning of the section. We explicitly state the use of the Kaplan–Meier estimator for survival analysis. Furthermore, we have clarified that a two-way ANOVA (factorial design) was employed for all other parameters, specifying diet and temperature as the primary factors.

• In summary, this study is valuable and the work is commendable, but clarification on the rearing protocol, diet, temperature conditions, and statistical analysis is essential to strengthen the manuscript, improve reproducibility, and increase its scientific impact. Thank you.

===================================================================================

Reviewer #3:

This study is very timely in the era of global climate change, as winters are becoming increasingly harsh and honey bee health is declining due to cold-season nutritional deficiencies. Supplementation with pre- and probiotics is therefore important. The study is well written and suitable for minor revision.

We sincerely thank you for this constructive critique and for recognizing the timeliness and relevance of this study in the context of global climate change. We have carefully considered each of your points and incorporated your recommended structural and conceptual changes into the revised manuscript. Detailed responses are provided below.

1. Lines 63–64: The claim that stress leads to bee population losses is overstated. Stressors can threaten colony health, but they do not necessarily cause population decline, particularly in managed honey bees, where resilience, husbandry practices, and nutrition mitigate impacts. Please temper this statement and, throughout the manuscript, avoid overgeneralizations by distinguishing colony-level stress from population-level trends. Support any population-level claims with appropriate, recent citations and revise the wording to reflect the current evidence base. As suggested, we have tempered our statements regarding population-level declines. We revised the manuscript to clearly distinguish between localized colony-level stress and broader population trends, explicitly acknowledging that managed bees often exhibit high resilience due to modern husbandry practices and supplemental nutrition. We have also added recent citations to support these nuanced points.

2. Hemagglutinins- check spelling it is the same spelling in the google. Done.

3. Line 68: Please insert a paragraph break to separate the oxidative stress discussion from the preceding text, improving readability and logical flow. Done.

4. In the final paragraph, please add a dedicated, well-structured section on honeybee–thermal–microbiome interactions. Summarize how the gut microbiome responds under thermal stress (both heat waves and cold spells), and how these shifts may influence resilience, colony health, and pathogen susceptibility. Highlight mechanisms by which bees maintain their microbiome and how the microbiome may, in turn, bolster bee resilience. After this synthesis, transition to probiotics and prebiotics, explaining how they may directly or indirectly support the gut microbiome. To create a clear, “funnel-shaped” narrative: start with thermal stress impacts, then discuss bee–microbiome resilience mechanisms, and conclude with interventions (probiotics/prebiotics). Please incorporate and appropriately cite relevant studies, for example:

• High temperatures: “Hot and Bothered: Bees’ Gut Microbiome Shifts Under Thermal Stress and Pathogen Infection” (J.I. Van Wyk et al., 2025); “Thermal niches of specialized gut symbionts: the case of social bees” (T.J. Hammer, E. Le, N.A. Moran).

• Low temperatures: “High abundance of lactobacilli in the gut microbiome of honey bees during winter” (Brar et al., 2025); “Overwintering Honey Bee Col

Attachment Submitted filename: Probiotics and prebiotics. Response to Reviewers.Final.docx

# Peer review: Decision Letter 1

**DOI:** 10.1371/journal.pone.0352149.r003

**Authors:** Vicente Martínez López

8 Jun 2026

<p>Protecting honey bees (Apis mellifera) from thermal stress: Probiotics and prebiotics buffer the survival and antioxidant enzyme activity

PONE-D-26-03238R1

Dear Dr. Bahreini,

We’re pleased to inform you that your manuscript has been judged scientifically suitable for publication and will be formally accepted for publication once it meets all outstanding technical requirements.

Within one week, you’ll receive an e-mail detailing the required amendments. When these have been addressed, you’ll receive a formal acceptance letter and your manuscript will be scheduled for publication.

An invoice will be generated when your article is formally accepted. Please note, if your institution has a publishing partnership with PLOS and your article meets the relevant criteria, all or part of your publication costs will be covered. Please make sure your user information is up-to-date by logging into Editorial Manager at Editorial Manager® and clicking the ‘Update My Information' link at the top of the page. For questions related to billing, please contact billing support.

If your institution or institutions have a press office, please notify them about your upcoming paper to help maximize its impact. If they’ll be preparing press materials, please inform our press team as soon as possible -- no later than 48 hours after receiving the formal acceptance. Your manuscript will remain under strict press embargo until 2 pm Eastern Time on the date of publication. For more information, please contact onepress@plos.org.

Kind regards,

Vicente Martínez López

Academic Editor

PLOS One

Additional Editor Comments (optional):

Reviewers' comments:

Reviewer's Responses to Questions

**Comments to the Author**

1. If the authors have adequately addressed your comments raised in a previous round of review and you feel that this manuscript is now acceptable for publication, you may indicate that here to bypass the “Comments to the Author” section, enter your conflict of interest statement in the “Confidential to Editor” section, and submit your "Accept" recommendation.

Reviewer #1: All comments have been addressed

Reviewer #2: All comments have been addressed

Reviewer #3: All comments have been addressed

**********

2. Is the manuscript technically sound, and do the data support the conclusions?

The manuscript must describe a technically sound piece of scientific research with data that supports the conclusions. Experiments must have been conducted rigorously, with appropriate controls, replication, and sample sizes. The conclusions must be drawn appropriately based on the data presented.

Reviewer #1: Yes

Reviewer #2: Yes

Reviewer #3: Yes

**********

3. Has the statistical analysis been performed appropriately and rigorously?

Reviewer #1: Yes

Reviewer #2: Yes

Reviewer #3: Yes

**********

4. Have the authors made all data underlying the findings in their manuscript fully available?

The PLOS Data policy requires authors to make all data underlying the findings described in their manuscript fully available without restriction, with rare exception (please refer to the Data Availability Statement in the manuscript PDF file). The data should be provided as part of the manuscript or its supporting information, or deposited to a public repository. For example, in addition to summary statistics, the data points behind means, medians and variance measures should be available. If there are restrictions on publicly sharing data—e.g. participant privacy or use of data from a third party—those must be specified.

Reviewer #1: Yes

Reviewer #2: Yes

Reviewer #3: Yes

**********

5. Is the manuscript presented in an intelligible fashion and written in standard English?

PLOS ONE does not copyedit accepted manuscripts, so the language in submitted articles must be clear, correct, and unambiguous. Any typographical or grammatical errors should be corrected at revision, so please note any specific errors here.

Reviewer #1: Yes

Reviewer #2: Yes

Reviewer #3: Yes

**********

6. Review Comments to the Author

Please use the space provided to explain your answers to the questions above. You may also include additional comments for the author, including concerns about dual publication, research ethics, or publication ethics. (Please upload your review as an attachment if it exceeds 20,000 characters)

Reviewer #1: The authors have revised the manuscript according to the Reviewr´s suggestions. They have done a a good work.

Reviewer #2: The authors have carefully and thoroughly addressed all the comments and concerns raised during the previous review round. Their responses are clear, detailed, and scientifically sound. The requested clarifications and revisions have been appropriately incorporated into the revised manuscript, which has significantly improved its clarity, quality, and scientific rigor. I appreciate the effort made by the authors to address the reviewers' suggestions and to strengthen the manuscript accordingly. In my opinion, the revised version satisfactorily addresses all major concerns, and I therefore recommend its acceptance for publication.

Reviewer #3: The manuscript has been significantly improved. No further comments.

The manuscript has been significantly improved. No further comments.

**********

7. PLOS authors have the option to publish the peer review history of their article (what does this mean?). If published, this will include your full peer review and any attached files.

If you choose “no”, your identity will remain anonymous but your review may still be made public.

**Do you want your identity to be public for this peer review?**  For information about this choice, including consent withdrawal, please see our Privacy Policy.

Reviewer #1: **Yes:** Maria Agustina Rodriguez

Reviewer #2: **Yes:** KHEDIDJI Hassiba

Reviewer #3: No

**********

# Editor report: Acceptance letter

**DOI:** 10.1371/journal.pone.0352149.r004

**Authors:** Vicente Martínez López

PONE-D-26-03238R1

PLOS One

Dear Dr. Bahreini,

I'm pleased to inform you that your manuscript has been deemed suitable for publication in PLOS One. Congratulations! Your manuscript is now being handed over to our production team.

At this stage, our production department will prepare your paper for publication. This includes ensuring the following:

* All references, tables, and figures are properly cited

* All relevant supporting information is included in the manuscript submission,

* There are no issues that prevent the paper from being properly typeset

You will receive further instructions from the production team, including instructions on how to review your proof when it is ready. Please keep in mind that we are working through a large volume of accepted articles, so please give us a few days to review your paper and let you know the next and final steps.

Lastly, if your institution or institutions have a press office, please let them know about your upcoming paper now to help maximize its impact. If they'll be preparing press materials, please inform our press team within the next 48 hours. Your manuscript will remain under strict press embargo until 2 pm Eastern Time on the date of publication. For more information, please contact onepress@plos.org.

You will receive an invoice from PLOS for your publication fee after your manuscript has reached the completed accept phase. If you receive an email requesting payment before acceptance or for any other service, this may be a phishing scheme. Learn how to identify phishing emails and protect your accounts at https://explore.plos.org/phishing.

If we can help with anything else, please email us at customercare@plos.org.

Thank you for submitting your work to PLOS ONE and supporting open access.

Kind regards,

PLOS ONE Editorial Office Staff

on behalf of

Dr. Vicente Martínez López

Academic Editor

PLOS One
